# Biochemical and Physicomechanical Cues of Biomaterials Guide Osteogenic Differentiation of Mesenchymal Stem Cells

**DOI:** 10.3390/ijms27156753

**Published:** 2026-07-28

**Authors:** Bofeng Pan, Adam Maalal, Dake Hao

**Affiliations:** 1 Department of Surgery, University of California Davis, Sacramento, CA 95817, USA; bfpan@health.ucdavis.edu (B.P.); amaalal@ucdavis.edu (A.M.); 2 Shriners Children’s Northern California, Sacramento, CA 95817, USA

**Keywords:** biomaterials, mesenchymal stem cells, osteogenic differentiation, bone tissue engineering, biochemical and physicomechanical stimuli

## Abstract

Bone regeneration remains a significant clinical challenge, particularly for large or critical-sized defects caused by trauma, disease, or congenital abnormalities. Mesenchymal stem cells (MSCs) have emerged as a promising cell source for bone tissue engineering, with their osteogenic differentiation playing a crucial role in bone repair. Biomaterials serve as scaffolds that facilitate MSC-mediated bone regeneration by providing structural support and mimicking the extracellular matrix (ECM). This review explores recent advancements in biomaterials designed to promote MSC osteogenesis through two primary approaches: biochemical and physicomechanical stimuli. Therapeutic agent-loaded scaffolds, incorporating growth factors, small molecules, gene materials, peptides, proteins, and extracellular vesicles (EVs), have been extensively studied for their ability to enhance osteogenic differentiation. However, concerns regarding toxicity, off-target effects, and regulatory limitations have led to increasing interest in biomaterials that utilize physicomechanical cues such as stiffness, viscoelasticity, topography, porosity, and dynamic forces (shear stress, compression, vibration) as alternative or complementary strategies. Furthermore, the synergistic effects of multiple physicomechanical cues are being explored to regulate MSC behavior for promoting bone regeneration. This review discusses current challenges, emerging trends, and future directions in the development of next-generation biomaterials that integrate biochemical and physicomechanical approaches for clinical applications in bone repair and regeneration.

## 1. Introduction

Bone grafting remains widely used, with an estimated 2.2 million orthopaedic bone-grafting procedures performed annually worldwide [1]. Fracture nonunion also represents a substantial clinical burden, with population-based studies reporting overall nonunion rates of approximately 1.9% to 4.9%, although the incidence varies considerably according to fracture site, injury severity, patient characteristics, and study population [2,3,4]. The rising prevalence of osteoporosis (affecting over 41 million people worldwide) [5] further underscores the need for improved bone regeneration strategies. Current treatment options for bone regeneration primarily include autografts, allografts, and synthetic bone substitutes [6,7]. Autografts, the gold standard in bone repair, involve harvesting bone tissue from the patient’s own body, typically from the iliac crest [8]. While this approach offers superior biocompatibility and osteoinductive properties, it is associated with major drawbacks such as donor site morbidity, limited availability, and prolonged recovery time [9,10]. Allografts, derived from donor bone tissue, help avoid donor site complications but come with risks such as immune rejection, disease transmission, and incomplete integration with the host bone [11,12,13]. Synthetic bone substitutes, including ceramics, polymers, and metallic implants, provide customizable alternatives but often lack the necessary biological cues to promote effective bone regeneration [14]. However, some synthetic implants exhibit poor biodegradability, which may lead to prolonged inflammation and eventual implant failure [15]. Given these limitations, there is a pressing need for alternative bone regeneration strategies that overcome the shortcomings of conventional treatments. Since native bone healing is orchestrated by the extracellular matrix (ECM), biomaterial development has increasingly focused on replicating ECM composition and function [16,17]. The native bone ECM provides biochemical cues, including adhesion ligands and matrix-bound growth factors, while simultaneously offering physicomechanical regulation through stiffness gradients, viscoelastic relaxation, fibrillar alignment, and load-induced deformation, all of which together support and enhance bone regeneration [18,19,20]. By recreating the biochemical and mechanical guidance, engineered scaffolds shift from passive volumetric fillers to active regulators of cells adhesion, mechanotransduction, and lineage specification [21,22,23]. Therefore, bone tissue engineering has emerged as a promising approach for bone defect treatment [19,24].

Mesenchymal stem cells (MSCs) can differentiate into osteoblasts, chondrocytes, and other musculoskeletal lineages, making them valuable in bone repair applications [25,26]. However, precisely guiding MSC differentiation toward osteogenesis remains a challenge. Common strategies to induce MSC osteogenesis primarily rely on biochemical signaling, employing growth factors, small molecules, and gene therapies [27,28]. Key osteogenic factors such as bone morphogenetic protein-2 (BMP-2), vascular endothelial growth factor (VEGF), and transforming growth factor-beta (TGF-β) play essential roles in modulating MSC differentiation and bone formation [29,30,31], while small molecules like dexamethasone and statins, along with viral and nonviral gene delivery approaches, have been explored for more targeted modulation [32,33]. To enhance the precision of biochemical delivery, bioengineered scaffolds are designed as controlled-release platforms incorporating hydrogels, nanoparticles, and bioactive ceramics to improve drug stability and reduce systemic toxicity [34,35,36]. Additionally, hybrid scaffolds with functionalized peptides, extracellular vesicles (EVs), or embedded cells are emerging as platforms that synergize biochemical and cellular interactions to support osteogenesis [20,37,38,39]. Beyond biochemical cues, the physicomechanical properties of biomaterials play a crucial role in directing MSC fate, since native bone ECM not only delivers biochemical signals but also transmits structural and mechanical cues that regulate adhesion, proliferation, and lineage commitment [40]. Scaffolds are thus being engineered with tailored properties such as stiffness, viscoelasticity, porosity and topography to engage mechanotransduction pathways. Moreover, dynamic physicomechanical stimuli such as fluid shear stress, cyclic compression, and vibration have also been demonstrated to directly stimulate MSC osteogenic differentiation [41,42]. These physicomechanical stimuli activate cellular mechanotransduction pathways (e.g., integrin-mediated focal adhesions and RhoA–YAP/TAZ signaling) and engage mechanosensors such as Piezo1/2 and TRPV4 ion channels, triggering intracellular signaling cascades that regulate gene expression and cytoskeletal dynamics to stimulate MSC osteogenic differentiation [40,43,44,45]. Compared to biochemical factors, physicomechanical stimulation offers a drug-free strategy that is potentially safer, more localized, and longer-lasting. While individual physicomechanical cues have been well studied, recent research highlights the importance of their synergistic integration for effective bone regeneration [46]. Bone tissue undergoes continuous remodeling under combined compressive, shear, and tensile forces, and mimicking these dynamic conditions in biomaterials could better replicate in vivo microenvironment [47,48]. While biochemical strategies remain fundamental, their limitations underscore the growing importance of physicomechanical cues in biomaterial design. By integrating multiple mechanical regulatory factors, next-generation biomaterials hold the potential to address current clinical challenges, enhance therapeutic outcomes, and accelerate the translation of regenerative strategies into clinical practice.

Although numerous reviews have discussed biomaterial-based bone regeneration, many focus primarily on one design dimension, such as therapeutic factor-loaded scaffolds, scaffold composition, MSC osteogenic signaling, or physicomechanical cues [35,40,49,50,51,52,53,54]. However, bone repair is not governed by a single regulatory input. Instead, successful regeneration requires the coordinated interaction of biochemical signaling, matrix-derived mechanical regulation, immune remodeling, angiogenesis, scaffold degradation, and stage-specific tissue maturation [28,40,55]. Therefore, the central argument of this review is that next-generation osteogenic biomaterials should be understood not simply as carriers of osteogenic factors or mechanically supportive matrices, but as integrated microenvironment-regulating systems. In this framework, biochemical cues provide molecular instructions for osteogenic commitment, physicomechanical cues regulate MSC mechanotransduction and matrix organization, and osteoimmunomodulatory cues shape macrophage–MSC crosstalk, inflammation resolution, and vascularized bone formation. This integrated perspective distinguishes the present review from previous single-axis discussions and provides a more comprehensive design logic for developing biomaterials that better reflect the dynamic and multicellular nature of bone healing.

**Figure 1:**
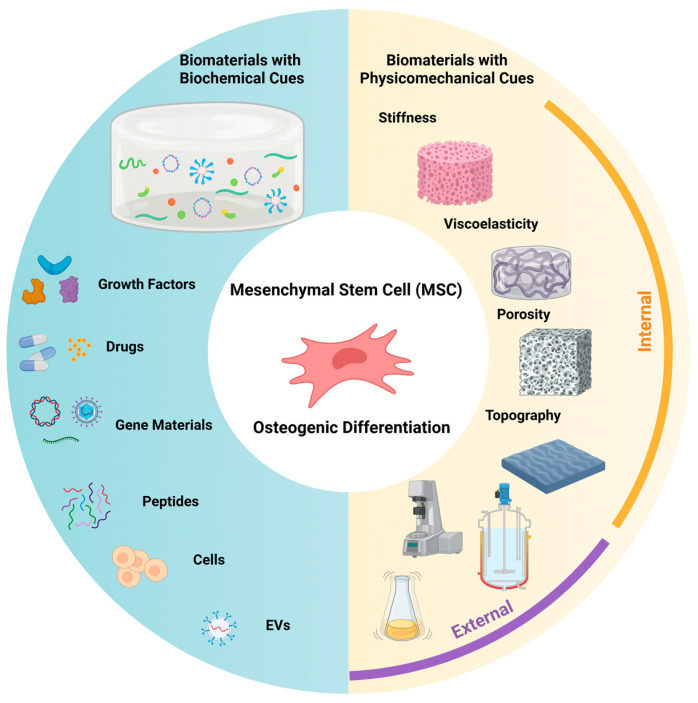
Schematic representation of biomaterial-based strategies for MSC differentiation and bone regeneration. Created in BioRender. Pan, B. (2026) https://BioRender.com/sob5g28 (accessed on 30 May 2026).

**Figure 2:**
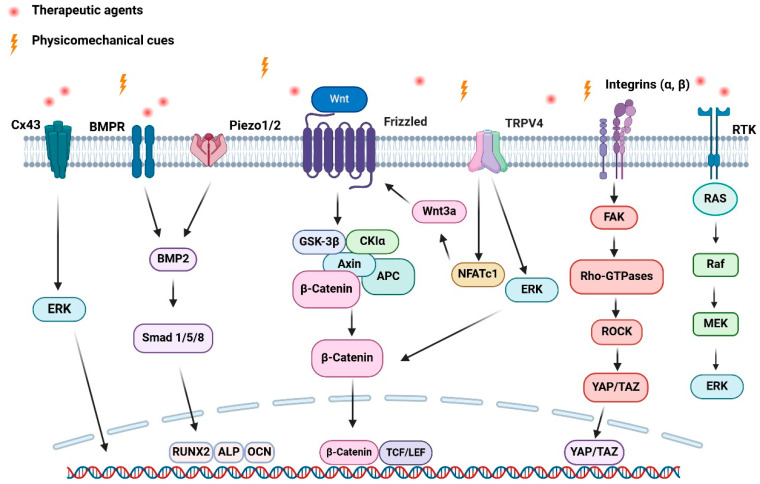
Integrated signaling pathways regulating biomaterial-guided MSC osteogenic differentiation. Created in BioRender. Pan, B. (2026) https://BioRender.com/xwnu3sh (accessed on 30 May 2026).

This review explores recent advances in biomaterials engineered to enhance MSC osteogenesis through biochemical, physicomechanical, and osteoimmunomodulatory cues (Figure 1), while further elucidating the molecular and signaling pathways involved in MSC fate regulation and osteogenic differentiation (Figure 2). First, we examine therapeutic agent-loaded scaffolds, including systems that deliver growth factors, small molecules, genetic material, bioactive peptides, cells, and extracellular vesicles to promote osteogenesis. Next, we discuss biomaterials that leverage structural and mechanical properties, such as stiffness, viscoelasticity, topography, porosity, and dynamic mechanical forces, to regulate MSC differentiation through mechanotransduction pathways. We then synthesize how multiple physicomechanical stimuli can act synergistically to create biomimetic microenvironments that better support bone regeneration. Importantly, we further introduce osteoimmunomodulation as an additional design axis, focusing on macrophage–MSC crosstalk and how biomaterial-derived biochemical and physicomechanical cues regulate macrophage polarization, inflammation resolution, angiogenesis, and MSC-mediated bone formation. We also discuss key translational considerations, including spatiotemporal control, degradation matching, mechanical durability, scalability, reproducibility, sterilization, and regulatory challenges. By shifting the discussion from single-cue optimization to coordinated microenvironment design, this review aims to provide a more instructive framework for next-generation osteogenic biomaterials and their clinical translation in bone tissue engineering.

## 2. Biochemical Cues in Biomaterial-Guided Osteogenesis

Traditional bone tissue engineering strategies often rely on biochemical stimulation of MSCs but achieving precise and sustained delivery remains a challenge [56,57]. In conventional approaches, bioactive factors are often administered in high doses, leading to rapid degradation, off-target effects, or systemic toxicity. Additionally, the absence of a controlled microenvironment can limit the efficiency of osteogenic differentiation [35]. Biomaterial-based scaffolds address these limitations by providing localized and sustained release of therapeutic agents to support MSC differentiation [18,27]. Strategies such as encapsulation of growth factors, controlled drug release, gene therapy, surface functionalization with bioactive peptides and proteins, and the incorporation of cells and extracellular vesicles (EVs) have been explored to enhance osteogenesis (Table 1) [58,59].

### 2.1. Growth Factors

Growth factors play an important role in regulating MSC differentiation and bone regeneration. Among them, bone morphogenetic proteins (BMPs), particularly BMP-2, are widely recognized for their potent osteoinductive capabilities. BMP-2 promotes MSC osteogenic differentiation primarily through the BMP/Smad pathway, where it binds to BMP receptors, triggering Smad1/5/8 phosphorylation and nuclear translocation to activate osteogenic genes like RUNX2, ALP, and OCN [30]. It also crosstalks with the Wnt/β-catenin and MAPK pathways, enhancing β-catenin stability and activating ERK1/2, JNK, and p38, further supporting osteoblast proliferation and differentiation (Figure 2) [60,61]. Through these interconnected pathways, BMP-2 effectively drives bone regeneration. Conventional BMP-2 delivery methods, such as direct soaking of scaffolds, suffer from rapid burst release, poor retention, and adverse effects, including ectopic bone formation and inflammatory responses [62]. To overcome these challenges, various biomaterial-based strategies have been developed to achieve controlled and sustained growth factor delivery.

One such approach involves biphasic calcium phosphate (BCP) scaffolds coated with a multilayer polymer film to regulate BMP-2 release. By incorporating an organosilicate-functionalized layer and a collagen/heparin-based film, this system significantly reduced the initial burst release while maintaining BMP-2 availability throughout the differentiation phase, leading to enhanced bone regeneration at minimal BMP-2 concentrations (0.01 mg/mL) [36]. Apart from BMP-2, BMP-7 has also been incorporated into biomaterials for osteogenesis. Like BMP-2, BMP-7 promotes MSC osteogenesis through the BMP/Smad pathway, activating RUNX2 and bone matrix proteins while also engaging PI3K/Akt and MAPK signaling to enhance osteoblast proliferation and differentiation (Figure 2). In the study of Mantripragada et al., to improve BMP-7 retention and bioactivity, chitosan microparticles have been employed for controlled BMP-7 release, which significantly enhanced bone density, collagen organization, and biomechanical properties while minimizing inflammation and uncontrolled bone formation [63].

While BMP-2 and BMP-7 have individually been explored for bone regeneration, studies suggest that their sequential delivery may further enhance osteogenesis. Yilgor et al. designed a nanoparticle-based system within polycaprolactone (PCL) scaffolds to achieve controlled BMP-2/BMP-7 release [64]. Their findings indicated that sequential delivery of BMP-2 followed by BMP-7 significantly improved rat bone marrow MSC differentiation, as evidenced by increased ALP activity and osteogenic marker expression. Given that bone regeneration requires both vascularization and osteogenesis, vascular endothelial growth factor (VEGF) has been widely investigated as a complementary factor. Casarrubios et al. investigated silicon-substituted hydroxyapatite (SiHA) scaffolds functionalized with VEGF to evaluate their osteogenic potential in an osteoporotic sheep model [65]. Their study demonstrated that VEGF-functionalized SiHA scaffolds significantly enhanced vascularization, increased osteoblast presence, and promoted thicker trabeculae compared to non-functionalized scaffolds.

Beyond BMPs and VEGF, other growth factors such as fibroblast growth factor-2 (FGF-2), platelet-derived growth factor (PDGF), and insulin-like growth factor-1 (IGF-1) have also been incorporated into biomaterials to enhance MSC osteogenesis and bone regeneration. FGF-2, in particular, has been investigated for its synergistic effects with BMPs in accelerating osteogenesis. Rambhia et al. developed a nanofibrous scaffold with controlled release of BMP-7 and FGF-2, demonstrating that optimized FGF-2 release kinetics improved BMP-7-induced bone formation by enhancing rabbit and human bone marrow stromal cells (BMSCs) recruitment, proliferation, and angiogenesis [66]. Similarly, a 3D-printed polycaprolactone/nano-hydroxyapatite (PCL/nHA) composite scaffold embedded with a collagen matrix has been developed for the controlled release of BMP-2 and FGF-2, demonstrating enhanced osteogenesis and vascularization [67]. Moreover, the co-delivery of BMP-7 and PDGF in mesoporous bioglass/silk fibrin scaffolds has shown promising results in facilitating hBMSCs recruitment, angiogenesis, and osteogenic differentiation, further reinforcing the importance of multi-factor delivery strategies [68]. Collectively, these studies highlight the potential of biomaterials as therapeutic agent-loaded scaffolds to achieve controlled and sustained growth factor release, effectively enhancing hBMSCs osteogenesis and bone regeneration while minimizing adverse effects.

Together, these studies suggest that the effectiveness of growth factor-loaded scaffolds depends less on simply increasing growth factor dose and more on controlling dose, localization, release kinetics, and delivery sequence. For potent osteoinductive factors such as BMP-2 and BMP-7, low-dose sustained release is generally preferable to burst release because it can reduce ectopic bone formation, inflammation, and other dose-related complications while maintaining osteogenic stimulation. For factors such as VEGF, FGF-2, and PDGF, timing is particularly important because early angiogenic or cell-recruiting signals must be coordinated with later osteogenic maturation. Therefore, growth factor delivery should be designed to first support recruitment and vascularization, and then provide sustained osteogenic signaling during later stages of bone formation. Clinically, this approach is relevant because it may reduce supraphysiological dosing, improve local retention, and increase safety compared with conventional growth factor delivery.

Biochemical cues, including growth factors, small molecules, gene materials, peptides, and extracellular vesicles, activate osteogenic pathways such as BMP/Smad and Wnt/β-catenin signaling. Physicomechanical cues, including stiffness, viscoelasticity, topography, porosity, and dynamic mechanical loading, regulate MSC fate through integrin/FAK, RhoA/ROCK, YAP/TAZ, Piezo1/2, TRPV4, and calcium-dependent signaling. These pathways interact through focal adhesion formation, cytoskeletal tension, calcium influx, nuclear mechanotransduction, and transcriptional crosstalk. Ultimately, they converge on osteogenic regulators and markers to promote osteogenic commitment, matrix deposition, and mineralization.

### 2.2. Small Molecules and Drugs

Small molecules and pharmaceutical agents have been explored as alternative or complementary strategies to growth factors for inducing MSC osteogenesis and bone regeneration. Compared to proteins, small molecules offer greater stability, cost-effectiveness, and easier integration into biomaterial-based delivery systems. Dexamethasone (Dex), a synthetic glucocorticoid, is widely used in MSC differentiation protocols due to its osteogenic properties. However, it also inhibits terminal osteoblast differentiation and promotes adipogenesis via PPARG upregulation, raising concerns about off-target effects. To mitigate these drawbacks, biomaterial-based controlled-release systems have been developed to regulate Dex dosage and prolong its bioactivity. Chen et al. developed dexamethasone-loaded biphasic calcium phosphate (BCP) nanoparticle/collagen porous composite scaffolds, which provided controlled Dex release for up to 35 days, which significantly enhanced hBMSC osteogenic differentiation and promoted bone regeneration in both in vitro and in vivo models [69]. Another study developed a 3D-printed biomimetic composite scaffold with sequential release of copper ions (Cu^2+^) and DEX to enhance both angiogenesis and osteogenesis [70]. The scaffold was composed of sodium alginate (SA), gelatin (GEL), and hydroxyapatite (HAP), with Cu^2+^ incorporated via cross-linking and DEX encapsulated in mesoporous silica nanoparticles (MSNs) for sustained release. The rapid release of Cu^2+^ facilitated early angiogenesis, while the gradual release of DEX promoted late-stage osteogenesis, mimicking the natural bone healing cascade.

Besides DEX, several other small molecules and drugs have been incorporated into biomaterials to enhance MSC osteogenesis and bone regeneration. Statins, commonly used for cholesterol regulation, have demonstrated significant osteogenic potential by stimulating BMP-2 expression and inhibiting osteoclast activity. A multilayered composite scaffold integrating β-tricalcium phosphate (β-TCP)/polycaprolactone (PCL) microchips with lovastatin-loaded nanofiber membranes was developed to achieve sustained statin release [71]. This system provided mechanical stability while supporting long-term drug delivery, promoting ALP activity, osteoblast proliferation, and new bone formation in osteoporotic rabbit models.

Additionally, lithium chloride (LiCl) has been shown to stimulate osteogenic differentiation by activating the Wnt/β-catenin signaling pathway (Figure 2) [72]. Incorporation of lithium into bioactive glass scaffolds has demonstrated enhanced bone regeneration in vitro and in vivo [73]. Similarly, Icariin, a flavonoid from Epimedium species, has been incorporated into polymeric scaffolds due to its ability to stimulate ERK and BMP pathways, leading to improved bone formation and healing [74].

These studies highlight the potential of small-molecule-loaded biomaterials in enhancing osteogenesis through controlled and sustained release. Compared with recombinant growth factors, small molecules generally offer better stability, lower cost, and easier incorporation into scaffolds, making them attractive for translational bone repair. However, many small molecules have broad biological activity and may influence multiple cell types or signaling pathways, which increases the risk of dose-dependent or off-target effects. Therefore, the priority design principle for small-molecule-based scaffolds is to maintain a therapeutic local concentration while avoiding systemic exposure or prolonged overstimulation.

### 2.3. Gene Delivery

Gene delivery has emerged as a powerful strategy for enhancing MSC osteogenesis and bone regeneration by directly modulating gene expression. Unlike growth factors and small molecules, genetic approaches offer the potential for sustained and precisely controlled osteoinductive signaling. Various gene delivery methods, including plasmid DNA (pDNA), mRNA, viral vectors and so on, have been explored to regulate key osteogenic pathways. These strategies target critical transcription factors and signaling molecules, such as RUNX2, BMPs, and Wnt/β-catenin, to promote bone formation (Figure 2). However, efficient and safe gene delivery remains a challenge, as naked nucleic acids are prone to degradation and exhibit poor cellular uptake. To address these limitations, biomaterial-based gene delivery systems, including nanoparticles, hydrogels, and polymeric scaffolds, have been developed to enhance transfection efficiency, prolong gene expression, and reduce off-target effects. These biomaterial platforms provide localized and sustained gene delivery, offering a promising alternative for controlled MSC differentiation and bone tissue engineering [75].

Among these approaches, viral vectors have been extensively utilized to enhance osteogenesis. For example, Bukharova et al. incorporated adenovirus-mediated BMP-2 gene therapy into gene-activated matrices (GAMs) to enhance MSC osteogenesis. Using an ex vivo approach, rabbit BMSCs were transduced with adenovirus-BMP2 (Ad-BMP2) before seeding into polylactide (PLA) granules and platelet-rich plasma (PRP)-based fibrin scaffolds. Compared to in vivo viral delivery, this method resulted in higher osteogenic marker expression, matrix mineralization, and bone formation. In a rat calvarial defect model, the ex vivo strategy achieved greater bone regeneration (33% vs. 28%) while minimizing immune responses, demonstrating the potential of biomaterial-assisted gene therapy for safer and more effective MSC osteogenesis [76]. Additionally, Zhang et al. developed silk fibroin scaffolds loaded with adenoviral BMP-7 (Ad-BMP7), achieving sustained BMP-7 release for up to 21 days [77]. These scaffolds enhanced osteogenesis by providing a continuous supply of BMP-7 while demonstrating favorable immunocompatibility, as pro-inflammatory cytokine levels returned to baseline after four weeks.

In addition to viral-based strategies, non-viral gene delivery systems have gained attention due to their lower immunogenicity and improved safety. Lackington et al. developed a non-viral gene delivery platform utilizing polyethyleneimine (PEI)-complexed pDNA encoding interleukin-1 receptor antagonist (IL-1Ra) within a collagen–hydroxyapatite (CHA) scaffold [78]. This system effectively protected MSCs from IL-1β-mediated inhibition of osteogenesis, mitigating inflammation and promoting bone regeneration. The gene-activated CHA scaffold not only provided structural support for MSC differentiation but also enabled sustained IL-1Ra expression, counteracting inflammatory bone loss. Similarly, Ding et al. designed a hydroxyapatite/chitosan microsphere (HA/CS-MS)-based gene-activated matrix (GAM) incorporating PEI-complexed pBMP2 pDNA [79]. This system exhibited high transfection efficiency, enabling sustained BMP-2 expression and promoting bone formation in vivo. The microsphere structure facilitated cell adhesion while ensuring controlled gene release over an extended period. Except for using pDNA as agent, Zhang et al. developed a chemically modified mRNA (cmRNA) encoding BMP-2 with improved osteogenic potential by optimizing its untranslated regions (UTRs) and incorporating 5-iodo modified pyrimidine nucleotides [80]. This cmRNA was delivered using transcript-activated matrices (TAMs) formed by loading it into collagen scaffold. In vitro, BMP-2 cmRNA significantly upregulated osteogenic and angiogenic gene expression in MSCs, while in vivo studies using a critical-sized femoral defect model in rats demonstrated superior bone formation and enhanced vascularization compared to previous BMP-2 delivery methods. This study highlights cmRNA as a promising alternative to conventional BMP-2 protein or gene therapy for bone regeneration. The study by Pan et al. developed a microRNA (miRNA)-activated hydrogel scaffold (MAHS) using 3D printing to enhance bone regeneration [81]. The researchers incorporated miR-29b, a key osteogenic regulator, into hydrogel scaffolds composed of gelatin and alginate. The scaffold degradation rate and miRNA release profile were systematically tuned to optimize osteogenesis. Among different formulations, the scaffold with an intermediate crosslinking degree exhibited the best balance between degradation and sustained miRNA release, significantly enhancing MSC differentiation and new bone formation both in vitro and in vivo. Together, these studies show that gene delivery is most effective when biomaterials protect genetic cargo, localize delivery, control expression duration, and limit off-target transfection. Compared with recombinant protein delivery, gene-based strategies can provide more sustained osteoinductive signaling, but their translation depends on vector safety, immune compatibility, transfection efficiency, and manufacturing reproducibility. Viral vectors offer high transduction efficiency but raise concerns regarding immunogenicity and regulatory complexity, whereas non-viral systems are generally safer but often less efficient. Therefore, the key design priority for gene-activated scaffolds is to balance potency and safety through spatially confined, temporally controlled, and immunologically compatible gene delivery.

### 2.4. Peptide-Modified Scaffold

Peptide-modified scaffolds have gained increasing attention in bone tissue engineering due to their ability to provide bioactive cues that enhance MSC adhesion, proliferation, and osteogenic differentiation. Peptides, as short amino acid sequences, can mimic functional domains of natural ECM proteins, enabling precise modulation of cellular behavior. Compared to full-length proteins, peptides offer advantages such as greater stability, lower immunogenicity, and easier incorporation into biomaterials. By functionalizing scaffolds with bioactive peptides, these materials can provide targeted biochemical signals that promote osteogenesis while maintaining structural integrity. For example, to improve the recruitment and regulation of endogenous cells in bone regeneration, Hao et al. developed a biomaterial functionalized with two synthetic peptides, LLP2A and LXW7, which selectively target integrins α4β1 and αvβ3, respectively [20,82,83,84,85,86]. These integrins play essential roles in mediating the adhesion of mesenchymal stem cells MSCs, osteoblasts, endothelial progenitor cells (EPCs), and endothelial cells (ECs), all of which contribute to osteogenesis and vascularization [83,87,88]. In vitro studies demonstrated that the LLP2A/LXW7-modified scaffold significantly enhanced the adhesion of these cell types, improving their recruitment and retention within the bone defect site. In an adult rat calvarial bone defect model, the peptide-functionalized scaffold promoted synergistic osteogenesis and angiogenesis, as evidenced by increased populations of osteoblasts, EPCs, and ECs. Similarly, in a fetal sheep spinal bone defect model, the scaffold augmented bone formation and vascularization without inducing adverse effects, demonstrating its safety and efficacy across different physiological environments. This peptide-modified biomaterial presents an off-the-shelf, biologically safe, and highly targeted approach for vascularized bone regeneration, addressing the limitations of conventional biomaterials by actively regulating specific endogenous cells. Additionally, incorporating peptide–amphiphiles (PAs) has emerged as a promising approach for enhancing osteogenesis within biomaterial scaffolds. For instance, a study developed a hybrid scaffold consisting of a collagen sponge reinforced with poly(glycolic acid) (PGA) fibers, functionalized with a PA nanofiber network containing the RGD (arginine–glycine–aspartic acid) motif [89,90]. These PA nanofibers mimic components and provide high surface area for MSC attachment, significantly improving cell adhesion and proliferation. When combined with perfusion culture, the PA-modified scaffold exhibited enhanced osteogenic differentiation of MSCs, as evidenced by increased ALP activity and osteocalcin expression. Moreover, in vivo implantation showed improved ectopic bone formation, suggesting that PA-functionalized scaffolds can provide dynamic and biomimetic environments that optimize MSC osteogenic potential.

In addition to scaffolds functionalized for MSC adhesion, peptide-modified biomaterials have also been designed as bioactive delivery systems for sustained release of osteoinductive peptides. For instance, Zhou et al. developed a 3D-printed silk-based scaffold functionalized with GHK-Cu, a copper-binding peptide, to enhance vascularized bone regeneration [91]. The scaffold was fabricated via polydopamine (PDA) coating, which enabled controlled release of GHK-Cu. This bioactive peptide enhanced MSC proliferation and osteogenic differentiation, while copper ions modulated macrophage polarization toward the M2 phenotype, reducing inflammation and stimulating angiogenesis. In addition, the scaffold stimulated transplanted BMSC proliferation and cytokine secretion, enhancing paracrine signaling and angiogenesis. In a critical-sized calvarial defect model, the scaffold supported superior vascularized bone repair, demonstrating its potential for bioactive ion-based biomaterial design.

Collectively, these studies show that peptide-functionalized biomaterials enhance osteogenesis and vascularization through more than one mechanism. On one hand, peptides provide biochemical specificity by activating receptor-mediated signaling, recruiting target cell populations, and delivering osteoinductive or immunomodulatory molecules [92,93]. On the other hand, adhesive peptides regulate cell attachment, focal adhesion formation, cytoskeletal tension, and mechanotransduction, thereby linking biochemical ligand presentation with physicomechanical sensing [94,95]. This dual function illustrates why biochemical and physicomechanical cues should not be viewed as completely independent scaffold parameters, but rather as interconnected design elements that jointly regulate MSC osteogenesis and bone regeneration.

### 2.5. Cells and EVs

Cell-based therapies and extracellular vesicle (EV)-functionalized biomaterials have emerged as promising approaches for enhancing MSC osteogenesis and bone regeneration. Unlike the approach mentioned above, which primarily relies on recruiting or inducing endogenous cells, this approach directly incorporates specific cell types into scaffolds to create a dynamic and biologically relevant microenvironment. MSCs, osteoblast progenitors, and other bone-related cell types can be incorporated into scaffolds to provide direct osteogenic support, promote angiogenesis, and modulate local immune responses. For example, Hsieh et al. transplanted enhanced green fluorescent protein (EGFP)-labeled porcine MSCs into calvarial defects of DsRed pigs using biomaterial scaffolds [39]. Through fluorescence imaging, the study demonstrated that MSC-seeded scaffolds facilitated the recruitment of host cells and significantly enhanced osteogenesis compared to acellular scaffolds. The findings further indicated that higher seeding densities promoted sustained MSC retention and greater bone regeneration, highlighting the importance of cell-seeded biomaterials in bone tissue engineering. Chen et al. investigated the efficacy of seeding human umbilical cord MSCs (hUCMSCs) and human bone marrow MSCs (hBMSCs) onto macroporous calcium phosphate cement (CPC) scaffolds for bone regeneration in a rat cranial defect model [96]. Both hUCMSC- and hBMSC-seeded scaffolds demonstrated significant increases in new bone formation and vascularization, highlighting the potential of MSC-loaded biomaterials for craniofacial and orthopedic applications. Alternatively, EVs, particularly exosomes derived from MSCs or immune cells, offer a cell-free approach by delivering osteoinductive proteins, microRNAs (miRNAs), short interfering RNAs (siRNAs), long non-coding RNAs (lnRNAs), and cytokines that enhance MSC differentiation and tissue repair. Compared to direct cell-based scaffolds, EV-based strategies eliminate the risks associated with immune rejection and tumorigenicity while still providing bioactive cues to stimulate osteogenesis and vascularization. One approach focuses on the controlled release of EVs from scaffolds to achieve prolonged bioactivity. For instance, Yang et al. developed a 3D-printed polydopamine (PDA)-functionalized gelatin/hyaluronic acid/nano-hydroxyapatite (Gel/HA/nHAP) scaffold to enable the controlled release of bone marrow mesenchymal stem cell-derived EVs (BMSC-EVs) for bone regeneration in diabetic rats [97]. The PDA modification improved EV retention and allowed gradual release, ensuring prolonged bioactivity. The study demonstrated that EVs derived from normal glucose-cultured BMSCs (NG-EVs) significantly enhanced BMSC migration, proliferation, and osteogenic differentiation compared to EVs derived from high glucose-cultured BMSCs (HG-EVs), highlighting the influence of diabetic conditions on EV function. In vivo experiments using a rat calvarial defect model further confirmed that the sustained release of NG-EVs from the scaffold promoted superior bone regeneration compared to both HG-EVs and scaffolds alone. Similarly, the study by Kang et al. developed an exosome-functionalized magnesium-organic framework (MOF)-based scaffold with osteogenic, angiogenic, and anti-inflammatory properties for accelerated bone regeneration [37]. This scaffold was designed to achieve a sustained release of human adipose-derived stem cell-derived exosomes (hADSCs-Exos), which were immobilized onto a poly(lactic-co-glycolic acid) (PLGA)/Mg-GA MOF composite scaffold. The slow release of exosomes from this scaffold provided prolonged bioactivity, enhancing osteogenic differentiation of human bone marrow-derived mesenchymal stem cells (hBMSCs), promoting angiogenesis in human umbilical vein endothelial cells (HUVECs), and mitigating inflammatory responses in RAW264.7 macrophages. In vivo experiments in a rat calvarial defect model confirmed that the scaffold facilitated superior bone regeneration, osseointegration, and vascularization. In contrast, other approaches involve directly immobilizing EVs onto scaffold surfaces without a controlled release mechanism. Xie et al. fabricated a decalcified bone matrix (DBM) scaffold coated with MSC-derived EVs to enhance angiogenesis and osteogenesis [98]. The study verified successful EV modification of DBM scaffolds using scanning electron microscopy and confocal imaging. When implanted into nude mice, the EV-modified scaffolds enhanced osteogenesis, as confirmed by micro-computed tomography and histological analysis. Immunohistochemical staining for CD31 further demonstrated increased vascularization, highlighting the crucial role of EVs in supporting angiogenesis during bone repair. In addition, Lazar et al. explored the use of matrix-bound vesicles (MBVs) in biomaterial scaffolds, mimicking the natural ECM-EV interactions [99]. Unlike EV delivery methods, where vesicles are released over time, MBVs remain physically embedded within the bioscaffold, ensuring prolonged bioactivity at the defect site. This strategy enhances EV retention, maintains localized therapeutic effects, and improves scaffold integration with host tissues. The use of MBV-functionalized scaffolds presents a promising avenue for achieving long-term EV-mediated bone regeneration while avoiding the challenges associated with rapid EV degradation. Together, these studies highlight the potential of cell- and EV-based strategies in bone tissue engineering. While cell-seeded scaffolds provide direct osteogenic and angiogenic support, EV-functionalized scaffolds offer a cell-free alternative with controlled or immobilized delivery of regenerative signals. Biomaterial design plays a crucial role in optimizing these approaches by ensuring cell viability, prolonging EV bioactivity, and facilitating targeted regenerative effects within bone defects. From a design perspective, cell- and EV-based scaffolds should be selected based on the clinical problem rather than used as broadly interchangeable regenerative tools. Cell-seeded scaffolds may be advantageous when direct osteogenic and paracrine support is needed, but they face challenges related to donor variability, survival after implantation, storage, immune compatibility, and manufacturing reproducibility. EV-functionalized scaffolds provide a more cell-free and potentially safer alternative, but their therapeutic effect depends strongly on EV source, culture conditions, loading efficiency, retention, and preservation of bioactivity. Therefore, the priority for EV-based biomaterials is not only to demonstrate osteogenic activity, but also to define reproducible EV potency, scalable production, and controlled presentation within the scaffold. This is particularly important for clinical translation because small changes in donor condition, disease state, or EV isolation method may substantially alter therapeutic outcomes.

Taken together, biochemical strategies are most effective when biomaterials provide localized, sustained, and stage-appropriate presentation of osteogenic signals rather than simple bolus delivery. Growth factors such as BMPs and VEGF offer strong osteoinductive or angiogenic activity, but their clinical use is limited by high-dose requirements, rapid diffusion, ectopic tissue formation, inflammation, and regulatory concerns. Small molecules provide greater stability and lower cost, but their effects are often less specific and may depend strongly on dose and release kinetics. Gene delivery can prolong osteogenic signaling, yet safety, transfection efficiency, and regulatory complexity remain major translational barriers. Peptide-functionalized scaffolds offer a more modular and chemically defined approach for regulating adhesion, recruitment, and signaling, while cells and extracellular vesicles provide broader paracrine regulation but introduce challenges related to manufacturing, storage, potency, and reproducibility. Therefore, the key design principle for biochemical biomaterials is not simply to incorporate more bioactive agents, but to control their dose, timing, localization, and interaction with the scaffold microenvironment to enhance osteogenesis while minimizing adverse effects.

### 2.6. Translational Challenges of Biochemical Cues

Although biochemical cues have been widely used to promote MSC osteogenesis, their clinical translation remains limited by several important factors. Growth factors such as BMPs and VEGF are highly potent, but their therapeutic effects depend strongly on local concentration, release kinetics, and retention within the defect site [100]. In practice, these molecules often suffer from short half-life, rapid diffusion, burst release, and loss of bioactivity after implantation, which can require supraphysiological dosing and increase the risk of ectopic bone formation, inflammation, edema, or other off-target responses [101]. Even when controlled delivery systems are incorporated, the manufacturing cost, storage instability, and regulatory burden associated with recombinant proteins continue to hinder broad clinical adoption [102].

Preclinical studies have shown that biochemical delivery can improve bone repair in small-animal models, but these outcomes are not always reproduced in more clinically relevant settings [103]. One major limitation is the mismatch between scaffold mechanics and the host bone environment. Many biomaterial systems perform well under simplified in vitro conditions but may fail to maintain sufficient load-bearing capacity, fatigue resistance, or structural integrity during degradation once implanted in vivo [104]. This issue becomes more pronounced in large defects, where vascularization is often insufficient and the mechanical demands are much higher than in standard cell culture or small rodent models [105]. Consequently, the predictive value of simplified in vitro assays remains limited, and validation in large-animal or other mechanically relevant defect models is essential before clinical translation.

These limitations also apply to other biochemical strategies, including small molecules, gene delivery, peptides, and extracellular vesicles. Although small molecules are generally more stable and economical than proteins, they often have broader biological activity and therefore require careful control of dose and timing to avoid off-target effects [106]. Gene-based approaches may provide longer-lasting osteoinductive signaling, but their translation is constrained by transfection efficiency, vector safety, immune compatibility, reproducibility, and regulatory complexity [107]. Similarly, peptide-functionalized and EV-based scaffolds offer more targeted bioactivity, yet their practical use still depends on reliable loading, preservation of function, and scalable manufacturing [108,109]. Together, these challenges underscore the need for alternative or complementary design strategies. In this context, physicomechanical cues offer a compelling route because bone is intrinsically a load-bearing tissue, and mechanical signals are fundamental regulators of MSC fate, matrix deposition, and regenerative remodeling. Compared with soluble biochemical cues, physicomechanical stimuli are generally more stable, localized, and less dependent on fragile cargo retention, making them well suited for long-term control of osteogenesis [110].

**Table 1:** Biochemical Cues in Biomaterial-Guided MSC Differentiation and Bone Regeneration.

Agent Types	Bioreactive Molecules	Biomaterials	Outcomes	Refer.
**Growth Factors**	BMP-2	Biphasic calcium phosphate (BCP) with A-PSQ/heparin/ multilayer coating	Controlled BMP-2 release significantly enhanced bone regeneration at very low BMP-2 concentrations by reducing initial burst release.	[36]
	BMP-7	Chitosan microparticles	BMP-7-loaded chitosan microparticles significantly promoted bone formation, increased bone porosity, and enhanced collagen fibril bundling.	[63]
	VEGF	Silicon-substituted hydroxyapatite (SiHA) macroporous scaffolds	VEGF-functionalized SiHA scaffolds significantly stimulated bone regeneration, increased osteoblast presence and promoted angiogenesis.	[65]
	BMP-2 and BMP-7	PCL scaffolds with PLGA/PHBV nanoparticle delivery system	Sequential delivery of BMP-2 and BMP-7 significantly enhanced osteogenic differentiation and increased ALP activity of MSCs compared to single or simultaneous delivery.	[64]
	BMP-7 and FGF-2	PLLA nanofibrous scaffolds	Individually controlled BMP-7 and FGF-2 release significantly enhanced bone regeneration by improving stem cell recruitment, proliferation, and angiogenesis.	[66]
	BMP-2 and FGF-2	3D-printed PCL/nano-HA/collagen composite scaffolds	Collagen-patterned scaffolds loaded with BMP-2 and FGF-2 significantly enhanced endogenous bone regeneration and angiogenesis.	[67]
	PDGF-B and BMP-7	Mesoporous bioglass/silk fibrin scaffolds	Dual delivery of PDGF-B and BMP-7 significantly enhanced bone regeneration in osteoporotic defects by promoting mesenchymal stem cell recruitment and osteogenic differentiation.	[68]
	IGF-1	3D-printed PLA/PEG/bredigite scaffolds coated with chitosan-PEG nanofibers	IGF-1-loaded scaffolds significantly enhanced bone regeneration, accelerated defect repair, and improved osteogenic differentiation of MSCs in vitro and in vivo.	[111]
**Small molecules and drugs**	Dexamethasone	3D-printed polycaprolactone (PCL) scaffolds with sustained dexamethasone release	Scaffold-mediated sustained dexamethasone release significantly enhanced osteogenic differentiation and mineralization.	[112]
	Dexamethasone	Biphasic calcium phosphate nanoparticles/collagen composite scaffold	Sustained release of DEX from scaffolds significantly promoted osteogenic differentiation of hMSCs and ectopic bone regeneration in vivo.	[69]
	Dexamethasone and copper ions (Cu^2+^)	3D-printed biomimetic composite scaffolds with mesoporous silica nanoparticles	Sequential release of copper ions and dexamethasone significantly enhanced osteogenesis, angiogenesis, and antibacterial activity for effective repair of large bone defects.	[70]
	Lovastatin	Multi-layered β-TCP/PCL composite scaffold with lovastatin-loaded nanofibers	Controlled release of lovastatin significantly promoted bone formation.	[71]
	Lithium chloride (LiCl)	Calcium phosphate cement (CPC) scaffold	Lithium chloride-loaded CPC scaffolds significantly accelerated bone regeneration and enhanced osteogenesis by activating the Wnt/β-catenin signaling pathway in osteoporotic bone defects.	[73]
	Icariin	3D-printed PTI composite scaffold	Icariin-incorporated scaffolds significantly promoted osteogenesis, angiogenesis, and bone regeneration.	[74]
**Gene Therapy**	Adenoviral vector carrying BMP2	gene-activated matrices (GAMs) composed of polylactide granules (PLA)	Ex vivo BMP2 delivery using transduced MSCs significantly enhanced osteogenesis, matrix mineralization, and bone formation compared to direct in vivo gene delivery.	[76]
	Adenoviral vector carrying BMP7	Silk fibroin scaffold	BMP7 adenovirus-loaded scaffolds sustained BMP7 release for up to 21 days, significantly enhancing osteogenesis and new bone formation.	[77]
	PEI-pDNA vector carrying IL-1Ra	Collagen–hydroxyapatite (CHA) scaffold	IL-1Ra gene-activated scaffolds protected BM-MSCs from IL-1β-mediated inhibition, preserved osteogenesis, and enhanced mineralization under inflammatory conditions.	[78]
	Chemically modified BMP-2 mRNA	Collagen sponges	BMP-2 mRNA-loaded scaffolds significantly enhanced osteogenesis and vascularization.	[80]
	PEI/pBMP2 plasmid DNA	Hydroxyapatite/chitosan microspheres (HA/CS-MS)	Gene-activated microspheres enabled sustained BMP-2 expression, significantly promoting osteogenesis and enhancing bone regeneration.	[79]
	miRNA (miR-29b)	3D-printed gelatin–alginate hydrogel scaffold	miR-29b-loaded scaffolds significantly enhanced osteogenesis by promoting MSC differentiation and accelerating new bone formation through sustained miRNA release.	[81]
**Peptides**	LLP2A and LXW7 integrin-targeting peptides	Collagen-based scaffold	LLP2A/LXW7-modified scaffolds significantly enhanced MSC adhesion, osteogenesis, and vascularization by promoting integrin α4β1 and αvβ3-mediated cell recruitment.	[20]
	Peptide-amphiphile	Collagen sponge reinforced with poly(glycolic acid) (PGA) fiber incorporation	Peptide-amphiphile nanofibers in a hybrid scaffold significantly enhanced MSC osteogenic differentiation and ectopic bone formation.	[89]
	Copper-binding peptide (GHK-Cu)	3D-printed silk fibroin scaffolds coated with polydopamine	GHK-Cu-modified scaffolds significantly enhanced vascularized bone regeneration by promoting MSC proliferation, osteogenesis, and M2 macrophage polarization.	[91]
**Cells and EVs**	Allogenic EGFP+ pMSCs	Hemostatic gelatin sponge scaffold	Transplanted MSCs recruited host cells, significantly enhanced osteogenesis, and promoted bone formation.	[39]
	hUCMSCs and hBMSCs	Macroporous calcium phosphate cement (CPC)	hUCMSC- and hBMSC-seeded CPC scaffolds significantly enhanced new bone formation and angiogenesis	[96]
	BMSC-EVs	3D-printed gelatin/hyaluronic acid/nano-hydroxyapatite (Gel/HA/nHAP) scaffolds	Sustained release of BMSC-EVs significantly enhanced bone regeneration, osteogenesis, and angiogenesis.	[97]
	MSC-EVs	Decalcified bone matrix (DBM) scaffold	EV-functionalized DBM scaffolds significantly enhanced vascularization and bone regeneration	[98]
	hADSCs-Exos	PLGA/Mg-GA metal–organic framework (MOF) scaffold	Exosome-functionalized MOF scaffolds significantly enhanced osteogenesis, angiogenesis, and anti-inflammatory responses	[37]

## 3. Physicomechanical Cues in Biomaterial-Guided Osteogenesis

Biochemical signaling has been extensively validated as an effective approach in promoting MSC osteogenic differentiation and bone regeneration. Growth factors, small molecules, gene materials, and bioactive peptides provide essential osteoinductive cues by activating key signaling pathways such as BMP/Smad, Wnt/β-catenin, and MAPK. These strategies have demonstrated significant success in preclinical and clinical applications, enhancing bone formation by modulating cellular behavior at the molecular level. Biomaterial-based delivery systems have further improved the efficacy of biochemical signals by enabling localized, sustained release and reducing off-target effects.

However, despite these advances, biochemical approaches face several limitations. Many growth factors, such as BMP-2, require high doses to achieve therapeutic effects, leading to concerns about ectopic bone formation, immune responses, and potential regulatory restrictions. The FDA has contra-indicated the use of these morphogenetic factors in skeletally immature patients due to concerns regarding toxicity, abnormal bone growth, and the potential for tumorigenesis [113,114]. In spinal bone defects, such as myelomeningocele (MMC), the administration of exogenous BMPs must be strictly avoided, as any abnormal bone formation could cause neural compression and worsen clinical symptoms [102]. Similarly, small molecules and gene-based therapies may suffer from inconsistent bioavailability, degradation issues, or off-target effects, limiting their long-term clinical application. These safety concerns highlight the need for alternative approaches that do not rely on exogenous biochemical factors while still effectively stimulating bone regeneration.

Additionally, while biochemical signals play a crucial role in initiating osteogenesis, bone is a mechanically dynamic tissue, and the extracellular microenvironment significantly influences MSC fate through physical and mechanical cues. While cell-seeded tissue-engineered bone scaffolds have been widely studied, transplanted cells have shown limited long-term engraftment, reducing their effectiveness in forming new bone [115,116,117]. Instead, bone formation during embryogenesis is primarily driven by endogenous MSCs, which undergo coordinated chondrogenesis and osteogenesis. Additionally, vascularization is critical in maintaining the long-term viability of regenerated bone tissue, particularly for large defects such as spinal injuries or vertebral malformations [118]. Given these biological realities, engineering biomaterials with mechanical properties that mimic the natural bone microenvironment has become a compelling strategy. Biomaterial-based mechanical stimulation has emerged as a promising alternative or complementary approach to regulate MSC osteogenic differentiation. The natural bone ECM provides not only biochemical cues but also structural and mechanical stimuli, such as stiffness, viscoelasticity, topography, and dynamic forces (e.g., shear stress, compression, vibration). These physicomechanical properties play a critical role in directing MSC lineage commitment, influencing cytoskeletal organization, and modulating mechanotransduction pathways. By engineering biomaterials with tailored mechanical properties, researchers aim to mimic the native bone microenvironment and create scaffolds that provide both structural support and bioactive mechanical stimuli for optimal bone regeneration (Table 2).

### 3.1. Internal Mechanical Stimulation

Physicomechanical stimuli can be categorized into internal mechanical properties inherent to biomaterials and external mechanical forces applied to the system. These stimuli play a crucial role in regulating MSC differentiation and bone regeneration through distinct mechanotransduction pathways. Internal mechanical stimulation refers to the physical characteristics of biomaterials, such as stiffness, viscoelasticity, topography, and porosity, which influence cell adhesion, cytoskeletal organization, and lineage commitment. These intrinsic material properties provide physicomechanical cues that mimic the natural bone ECM, directing MSC differentiation without the need for exogenous biochemical factors.

#### 3.1.1. Stiffness

Stiffness, defined as the resistance of a material to deformation, plays a fundamental role in regulating MSC behavior in bone tissue engineering. The ECM of native bone exhibits high mechanical stiffness, providing critical biophysical signals that guide osteogenic differentiation. MSCs respond to substrate stiffness through mechanosensitive pathways, including integrin clustering, cytoskeletal tension, and nuclear mechanotransduction, which collectively regulate lineage commitment. Biomaterials with tunable stiffness are widely explored for their ability to recapitulate the native bone microenvironment, enhance cell adhesion, and stimulate osteogenesis. Recent studies highlight that tunable stiffness hydrogels can guide MSC differentiation even in the absence of soluble osteogenic inducers. For example, a study investigating 3D ECM stiffness and MSC differentiation demonstrated that methacrylate gelatin (GelMA) hydrogels with higher stiffness (~25 kPa) activated PCK2 expression, leading to enhanced glycolysis and osteogenic differentiation. In contrast, softer matrices (~6 kPa) suppressed PCK2 activity, resulting in a metabolic shift that favored adipogenic differentiation. Mechanistically, PCK2 promoted glucose metabolism via phosphofructokinase (PFKP) activation, driving osteogenesis through AKT/ERK signaling (Figure 2) [119]. Similarly, a study on 3D-bioprinted alginate-gelatin (Alg-Gel) composite hydrogels demonstrated that stiffer matrices (225 kPa) enhanced osteogenic marker expression (ALP) compared to softer substrates (50 kPa), emphasizing that biophysical cues alone can significantly drive stem cell fate decisions [120]. However, excessive stiffness (>700 kPa) may impair cellular mechanosensing, limiting osteogenic differentiation. Studies on semi-flexible hydrogels suggest that dynamic stiffness adaptation—where hydrogels transition from a softer, cell-permissive state (~30–40 kPa) to a stiffer, bone-mimicking state (>100 kPa) over time—can enhance MSC migration toward stiffer regions, promote cytoskeletal tension via integrin-FAK signaling (Figure 2), and facilitate sustained osteogenic differentiation [121]. This approach prevents excessive mechanical stress that can arise from static, overly rigid substrates (>400 kPa), which are known to reduce focal adhesion turnover and hinder osteogenesis. This study demonstrated that collagen-fibrinogen crosslinked hydrogels initially allowed rapid MSC attachment in the soft phase (~50 kPa), followed by gradual stiffening (~200 kPa), which enhanced RUNX2 expression and bone matrix deposition. In addition to bulk stiffness modulation, recent advancements in localized stiffening strategies suggest that heterogeneous stiffening within hydrogel matrices can further accelerate MSC mechanosensing and osteogenic differentiation. A supramolecular hydrogel network incorporating acryloyl-functionalized nanoparticles (AcNPs) to create microscale stiffened regions was shown to enhance focal adhesion formation, cytoskeletal organization, and mechanotransduction, leading to increased osteogenic gene expression (RUNX2, ALP, OCN) (Figure 2). Compared to uniformly stiffened hydrogels, these spatially controlled mechanical gradients more effectively mimic the heterogeneous stiffness of native bone ECM, resulting in faster stem cell adhesion, spreading, and differentiation [122]. In addition to hydrogels, nanofiber-based scaffolds also offer tunable stiffness properties for bone tissue engineering. Recent studies have demonstrated that electrospun nanofibers with adjustable stiffness can effectively regulate MSC differentiation. A study using aligned ultrafine poly(L-lactic acid) (PLLA) nanofibers demonstrated that increasing fiber stiffness via annealing treatment enhanced MSC osteogenic commitment by upregulating RUNX2 expression and osteogenic differentiation markers (ALP, OCN) [123]. Mechanistically, stiffer nanofibers activated macrophage migration inhibitory factor (MIF)-mediated AKT/YAP signaling, which regulated MSC fate toward osteogenesis (Figure 2). These findings suggest that electrospun nanofiber scaffolds with tunable stiffness can serve as an effective alternative to hydrogel-based biomaterials in guiding MSC differentiation and promoting bone regeneration. Beyond polymeric nanofibers, a recent study by Wang et al. developed a silica (SiO_2_) nanofiber-chitosan (SiO_2_ NF-CS) composite scaffold with gradient stiffness and superelastic properties, mimicking the natural bone ECM stiffness transition [124]. In vitro, MSCs seeded on stiffened SiO_2_ NF-CS scaffolds exhibited enhanced FAK/YAP signaling activation, leading to higher expression of osteogenic markers (RUNX2, ALP, OCN) and increased mineralization (Figure 2). Similarly, in vivo rat cranial defect models demonstrated that stiffer nanofiber regions promoted greater bone formation, as confirmed by micro-CT imaging, histological staining, and qPCR analysis.

These studies indicate that stiffness-mediated osteogenesis is context-dependent and should not be interpreted as a simple “stiffer is better” design rule. Moderate-to-high stiffness can enhance integrin clustering, cytoskeletal tension, YAP/TAZ activation, and RUNX2-mediated osteogenic differentiation, but excessive stiffness may impair cell spreading, focal adhesion turnover, matrix remodeling, and immune compatibility. Therefore, the priority design principle is to provide sufficient mechanical support while maintaining a cell-permissive microenvironment. Dynamic or spatially heterogeneous stiffness may be particularly valuable because it can mimic the evolving mechanical environment of bone healing, with an initially softer matrix supporting cell attachment, migration, and infiltration, followed by gradual stiffening that promotes osteogenic mechanotransduction and matrix mineralization.

#### 3.1.2. Viscoelasticity

Bone tissue exhibits viscoelastic properties, meaning that its mechanical response is both time-dependent and rate-dependent, distinguishing it from purely elastic materials. The ECM of bone dissipates mechanical loads through stress relaxation and creep, influencing cell–matrix interactions and MSC differentiation. Recent studies suggest that viscoelasticity plays a crucial role in regulating cell adhesion, spreading, polarization, and lineage commitment, making it a key design consideration for biomaterials in bone tissue engineering. Unlike purely elastic scaffolds, which restrict cell motility and mechanosensing, viscoelastic biomaterials dynamically adapt to mechanical stimuli, facilitating integrin clustering, actomyosin contraction, and mechanotransduction signaling. Consequently, incorporating tunable viscoelasticity into biomaterial design offers new opportunities to optimize stem cell osteogenesis and bone regeneration. Hydrogels are regarded as promising biomaterials for mimicking bone ECM due to their highly tunable biophysical properties [125,126]. Chaudhuri et al. were the first to develop a synthetic hydrogel system specifically designed to simulate the stress relaxation behavior of viscoelastic tissues, providing a well-controlled platform for studying MSC mechanotransduction [127]. Their pioneering study demonstrated that osteogenic differentiation of MSCs is highly dependent on matrix viscoelasticity, as MSCs cultured on hydrogels with faster stress relaxation kinetics exhibited significantly enhanced osteogenic marker expression, compared to those on static, purely elastic substrates. This effect was attributed to rapid stress relaxation, which facilitated integrin adhesion, increased actomyosin contraction, and promoted the nuclear localization of the mechanosensitive transcriptional regulator YAP, collectively driving MSC osteogenesis (Figure 2). Furthermore, MSCs in rapidly relaxing hydrogels (initial modulus ~17 kPa) exhibited enhanced collagen-1 deposition and mineralization, reinforcing the critical role of matrix viscoelasticity in guiding osteogenic differentiation. Expanding on this, Whitehead et al. further demonstrated that viscoelastic alginate hydrogels with faster stress relaxation enhances MSC osteogenesis by promoting integrin-mediated mechanotransduction and YAP nuclear localization [128]. Additionally, MSC spheroids embedded in stress-relaxing hydrogels exhibited greater osteogenic differentiation and improved bone formation in rat calvarial defect models, even in the absence of osteoinductive growth factors like BMP-2. In addition, a study by Liu et al. developed a bone ECM-mimicking 3D-printed scaffold composed of poly(L-lactide) (PLLA) infused with a chitin whisker/chitosan (HCHW-CS) composite hydrogel, designed to replicate the liquid crystalline and viscoelastic nature of native bone ECM [129]. This scaffold exhibited tunable stress relaxation, which significantly enhanced MSC adhesion, spreading, and osteogenic differentiation. Furthermore, in vivo studies in rat models demonstrated that faster stress relaxation within the scaffold promoted bone formation, emphasizing the importance of viscoelastic biomaterials in optimizing MSC mechanotransduction and guiding bone regeneration. In addition to stress relaxation, molecular mobility within biomaterials has also emerged as a key factor influencing cellular mechanotransduction. A study by Álvarez et al. developed dynamic supramolecular polymers with enhanced molecular motion, demonstrating that increased scaffold mobility enhances β1-integrin activation, cytoskeletal organization, and downstream osteogenic signaling, indicating that beyond macroscopic viscoelastic properties, the molecular-scale mobility of biomaterials can further fine-tune cell adhesion, spreading, and differentiation [130]. Collectively, these studies suggest that viscoelasticity regulates MSC osteogenesis by controlling how cells dynamically remodel and mechanically interpret the surrounding matrix. Faster stress relaxation can promote integrin clustering, cell spreading, actomyosin contractility, YAP nuclear localization, collagen deposition, and mineralization, indicating that cells respond not only to initial stiffness but also to time-dependent matrix resistance. However, rapid stress relaxation should not be viewed as universally beneficial. If a scaffold relaxes or deforms too quickly, it may lose mechanical stability, reduce long-term structural support, or fail to maintain appropriate load transfer in bone defects. Therefore, the priority design principle for viscoelastic biomaterials is to balance cell-permissive matrix remodeling with sufficient mechanical retention.

#### 3.1.3. Topography

Surface topography plays a critical role in regulating MSC behavior and directing osteogenic differentiation in bone tissue engineering (BTE). Unlike modifications in stiffness and viscoelasticity, which may alter the bulk mechanical properties of a material, topographical structures can be precisely engineered on biomaterial surfaces with minimal impact on their overall mechanical integrity, making them highly clinically translatable. Various micro- and nanoscale surface features, including grooves, ridges, pits, nanotubes, and hierarchical hybrid structures, have been shown to influence cell adhesion, cytoskeletal organization, and mechanotransduction pathways, ultimately guiding MSC lineage commitment (Table 2). Since Harrison et al. first demonstrated in 1911 that stem cell differentiation could be influenced by substrate topography, extensive research has been conducted to optimize surface microstructures and nanotopographies to enhance osteogenic potential [131]. For example, Joo et al. developed a biomimetic scaffold integrating hydroxyapatite (HAp) with a polyvinylidene fluoride-co-trifluoroethylene (P(VDF-TrFE)) matrix, designed to use topographical roughness for enhanced osteogenesis and bone regeneration [132]. Their study demonstrated that increasing surface roughness, achieved through HAp incorporation, significantly improved MSC adhesion, proliferation, and differentiation. Notably, rougher surfaces (Ra ~15.65 nm) exhibited greater cell spreading and vinculin clustering, which are critical for focal adhesion maturation and mechanotransduction. Furthermore, in in vivo calvarial defect models, HAp/P(VDF-TrFE) scaffolds accelerated bone formation and increased bone volume fraction, confirming the osteoinductive potential of rough surfaces. These findings highlight that topographical roughness enhances mechanotransduction, influencing cytoskeletal tension and integrin signaling, which collectively drive MSC osteogenesis.

Recently, micropatterned and nanopatterned biomaterials have attracted significant attention due to their potential to enhance osteogenic differentiation and bone regeneration. Unlike bulk material modifications, these surface patterns provide localized mechanical cues that influence cell adhesion, cytoskeletal organization, and lineage commitment. For example, Abagnale et al. systematically investigated the effect of microgrooved polyimide substrates with varying ridge widths on MSC differentiation [133]. Their study revealed that ridge widths of 2 μm significantly enhanced osteogenic differentiation, whereas wider ridges (~15 μm) favored adipogenic differentiation, highlighting the importance of precise micropatterning in directing stem cell fate. The authors attributed this phenomenon to cytoskeletal tension and cell morphology, where elongated MSCs on narrower ridges preferentially committed to osteogenic lineage, while rounded cells on wider ridges exhibited adipogenic tendencies. These findings underscore the critical role of micropatterned surface topography in engineering osteoinductive biomaterials, reinforcing the potential of microstructured scaffolds in bone tissue engineering applications. For nanopatterns, Pedrosa et al. investigated the impact of precisely controlled silicon nanopillar arrays on osteogenic differentiation of human mesenchymal stem cells (hMSCs) [134]. Their study demonstrated that nanopillar diameter and spacing critically influenced hMSC fate, with larger-diameter nanopillars (100 nm) promoting osteogenic differentiation, while smaller pillars (50 nm) had limited effects. Additionally, the spacing between nanopillars played a role in age-dependent differentiation responses: younger donor-derived hMSCs responded better to smaller-spaced nanopillars (~140 nm), whereas older donor-derived MSCs exhibited enhanced osteogenesis on larger-spaced nanopillars (~200 nm). The researchers attributed this phenomenon to nanotopography-mediated integrin clustering and focal adhesion maturation, leading to enhanced RUNX2, COL1A1, and OCN expression (Figure 2). These findings highlight the importance of precise nanopattern engineering in developing biomaterials tailored for patient-specific bone regeneration applications. By combining nanopatterns and micropatterns, specifically designed biomaterials can precisely guide MSC differentiation and enhance osteogenesis through multiscale hierarchical surface modifications. For example, Zhao et al. fabricated hydroxyapatite (HA) bioceramics with a micro/nano hybrid surface structure, combining micropatterned features with nanorods, to investigate their impact on MSC differentiation [135]. Their study revealed that integrins played a crucial role in mediating cell adhesion and mechanotransduction, with micro- and nano-features activating different integrin subunits. Notably, micropatterned surfaces preferentially activated integrin α5β1, while nanorods promoted β1-integrin expression, suggesting distinct yet complementary pathways in topography-induced osteogenesis. Further, BMP-2 signaling and connexin-43 (Cx43)-mediated cell–cell communication were also upregulated in response to micro/nano hybrid structures, reinforcing the importance of multiscale hierarchical topographies in biomaterial design. Beyond bioceramics, 3D hierarchical nanofiber scaffolds have also emerged as a promising strategy to guide endogenous bone regeneration. For instance, Chen et al. developed a 3D scaffold composed of polycaprolactone (PCL) electrospun nanofibers with controlled structural hierarchy and nanotopography to investigate its role in osteogenesis and cranial bone regeneration [136]. This featured radially and vertically aligned nanofibers (RAS/VAS), which significantly enhanced bone marrow stem cell (BMSC) migration and upregulated bone-related gene expression in vitro. Notably, RAS scaffolds guided cell infiltration from the surrounding tissue into the defect site, while VAS scaffolds facilitated directional cell migration from the dura and periosteum. In in vivo rat calvarial defect models, RAS/VAS scaffolds significantly improved new bone formation, mineral density, and collagen fiber alignment compared to conventional scaffolds. Furthermore, the regenerated bone architecture closely mimicked the nanotopographical alignment of the scaffold, reinforcing the importance of topographical cues in guiding osteogenesis. These findings demonstrate that hierarchical 3D topographies can mimic native bone ECM, enhance osteogenic signaling pathways, and serve as an effective surface modification strategy for bone regeneration applications. These findings demonstrate that topographical modifications, including surface roughness, micropatterns, nanopatterns, and hierarchical structures, provide critical cues for MSC adhesion, mechanotransduction, and osteogenic differentiation.

#### 3.1.4. Porosity

Porosity is a fundamental design parameter in bone tissue engineering, directly influencing cell infiltration, nutrient diffusion, vascularization, and overall bone regeneration. A well-designed porous structure provides an optimal microenvironment for osteogenesis by facilitating cell adhesion, proliferation, and ECM deposition, while also ensuring adequate mechanical support for new bone formation [52,137,138]. Pore size, interconnectivity, and porosity levels must be carefully optimized to balance mechanical stability with biological functionality. For example, a study on 3D-printed porous scaffolds demonstrated that optimizing porosity levels (50–70%) and pore geometry significantly influenced mechanical properties and bone regeneration efficiency compared hexagonal, rectangular, curved, and zigzag pore architectures, finding that porous structures mimicking trabecular bone maintained sufficient mechanical strength while promoting osteoblast activity and mineralization [52,139]. Similarly, Feng et al. developed a lotus root-inspired biomimetic scaffold featuring multichannel interconnected porosity, designed to enhance both osteogenesis and angiogenesis 3D-printed scaffolds incorporated hollow channels, mimicking the natural structure of lotus roots, to improve nutrient exchange, oxygen diffusion, and fluid transport [140]. Their study demonstrated that bone marrow stem cells (BMSCs) adhered and proliferated more efficiently on these scaffolds due to enhanced surface area and interconnectivity. Furthermore, in vivo experiments using rat muscle and rabbit calvarial defect models showed that lotus root-like scaffolds significantly promoted blood vessel formation and accelerated bone defect healing, outperforming traditional scaffolds. Beyond pore size and geometry, recent reviews highlight the importance of hierarchical porosity, which integrates macropores (>300 μm), micropores (50–150 μm), and nanopores (<50 nm) into scaffold design to enhance both osteogenesis and vascularization [52]. Macropores primarily facilitate cell migration and vascularization, while micropores improve cell attachment and ECM deposition, and nanopores influence protein adsorption and integrin-mediated mechanotransduction. Additionally, interconnected porosity plays a crucial role in nutrient and oxygen transport, supporting long-term tissue integration. Advances in fabrication techniques, such as 3D printing, freeze-drying, and gas foaming, now enable precise control over pore architecture, allowing scaffolds to better mimic the native bone ECM and support large bone defect healing [141,142]. Overall, these studies suggest that porosity-mediated bone regeneration depends on balancing transport, tissue ingrowth, vascularization, and mechanical stability. Larger and more interconnected pores generally improve cell infiltration, nutrient diffusion, vascular invasion, and ECM deposition, but excessive porosity can reduce compressive strength, fatigue resistance, and long-term dimensional stability. Therefore, the priority design principle is not to maximize porosity, but to engineer hierarchical and interconnected pore networks that support early cell migration and angiogenesis while preserving sufficient mechanical support during bone formation.

### 3.2. External Mechanical Stimulation

External mechanical stimulation plays a crucial role in modulating MSC behavior and enhancing osteogenic differentiation in bone tissue engineering. In the native bone microenvironment, mechanical cues such as compression, tension, shear stress, and vibration influence bone remodeling and regeneration by activating mechanotransduction pathways. Static culture conditions sometimes fail to provide sufficient nutrient exchange and biomechanical cues, whereas dynamic mechanical stimulation, applied through bioreactors, better mimics physiological conditions, improving cell proliferation, differentiation, and ECM deposition [143]. Various mechanical forces have been widely explored for their ability to regulate MSC mechanosensing and enhance osteogenesis.

Bone matrix naturally experiences compressive loading due to physiological forces such as gravity and muscle contraction. Studies have demonstrated that compressive stress can enhance osteogenic differentiation of MSCs when applied at optimal magnitudes. For example, Yamada et al. investigated the effects of cyclic uniaxial compression on MSC-laden octacalcium phosphate–gelatin (OCP/Gel) scaffolds. Their findings revealed that a moderate compressive strain (20%) significantly upregulated osteogenic markers such as osteopontin and osteocalcin, whereas excessive strain (40–60%) inhibited differentiation [144]. This suggests that compressive stress must be carefully optimized to balance mechanical stimulation and cell viability. Moreover, the study highlighted that scaffold composition plays a critical role in mechanotransduction, as the biodegradable OCP/Gel scaffold facilitated mechanical stress transmission while promoting MSC adhesion and differentiation. These findings reinforce the importance of applying controlled compressive stress in scaffold design for effective bone regeneration.

Beyond compression, tensile stress is another essential mechanical force in bone physiology, directly influencing MSC differentiation and bone regeneration. Clinically, tensile stimulation mimics distraction osteogenesis, a surgical technique in which gradual stretching promotes new bone formation across fracture gaps by stimulating cellular mechanotransduction pathways. In vitro studies using tensile strain bioreactors have demonstrated that MSC osteogenesis can be effectively enhanced by carefully optimizing loading parameters, including strain magnitude, frequency, and duration. For example, a study investigated the effects of cyclic mechanical strain (0.5 Hz, 2000 με) on MSC proliferation and differentiation showed that bone-related transcription factors and growth factors, including RUNX2, Ets-1, TGF-β, bFGF, IGF-II, and ALP, were significantly upregulated under tensile loading, indicating that tensile stress directly enhances MSC osteogenesis [145]. Additionally, the study confirmed that mechanical stretching stimulated [137,138] mineralization and collagen deposition, reinforcing the role of tensile stress in promoting bone matrix formation.

In addition to direct mechanical loading, fluid shear stress is another important factor in modulating MSC behavior and enhancing osteogenesis. Unlike static culture conditions, where nutrient diffusion is often limited, dynamic fluid flow promotes uniform cell distribution, improves oxygen and nutrient transport, and facilitates metabolic waste removal. As a result, bioreactor-based fluid shear stimulation has emerged as a promising strategy to enhance MSC osteogenic differentiation and optimize scaffold-based bone regeneration. Among the different approaches, Yamada et al. demonstrated that fluid shear stress alone, even without chemical osteoinductive supplements, can direct MSC differentiation toward an osteogenic lineage [146]. Using a laminar flow bioreactor, the study applied low-magnitude fluid shear stress to bone marrow stromal cells (BMSC) cultured on 3D polyester-based microporous scaffolds. Their findings revealed that fluidic stimulation upregulated RUNX2 expression, enhanced F-actin polymerization, and promoted ROCK1-mediated mechanotransduction (Figure 2), leading to increased collagen type 1 deposition and calcium mineralization within the scaffold. These results underscore the mechanosensitive nature of MSCs, where controlled fluid shear forces alone can initiate osteogenesis without requiring additional biochemical cues. The findings highlight the potential of fluid-flow-driven bioreactors as a non-invasive, efficient method to induce osteogenesis in MSCs. By fine-tuning fluid dynamics, scaffold architecture, and culture conditions, shear stress can be strategically utilized to enhance bone tissue engineering outcomes.

Similarly, low-magnitude, high-frequency vibration plays a crucial role in maintaining bone homeostasis and promoting MSC osteogenic differentiation. Recent studies have demonstrated that vibrational stimulation can enhance calcium deposition, type I collagen expression, and osteogenic gene upregulation, highlighting its potential for bone tissue engineering (BTE). For example, Campsie et al. developed a nanovibrational bioreactor capable of delivering highly controlled nanoscale mechanical stimulation to MSCs at a frequency of 1 kHz and 30 nm displacement [147]. This system demonstrated that osteogenic differentiation could be induced purely through nanovibrational stimulation, without requiring biochemical osteoinductive factors. Mechanistically, vibration-mediated mechanotransduction promoted focal adhesion maturation and cytoskeletal tension, leading to enhanced expression of osteogenic markers such as RUNX2, OPN, and ALP (Figure 2). Additionally, atomic force microscopy (AFM) confirmed that osteogenesis resulted directly from vibrational stimuli, rather than strain hardening of the collagen matrix. This study underscores the potential of nanovibration as a scalable, non-invasive strategy for MSC-driven bone regeneration. External mechanical stimulation is a key regulator of MSC fate in bone tissue engineering, with compression, tension, shear stress, and vibration each contributing unique mechanotransductive cues that enhance osteogenic differentiation. By integrating precisely controlled mechanical forces into bioreactor systems, researchers can optimize MSC mechanosensing, ECM production, and scaffold integration, making mechanical stimulation a powerful tool for enhancing bone regeneration strategies.

### 3.3. Synergistic Effect of Physicomechanical Cues

The osteogenic differentiation of MSCs is not controlled by a single mechanical factor but rather by a synergistic interplay of multiple cues within the three-dimensional ECM. In vivo, MSCs are simultaneously exposed to hydrostatic pressure, fluid shear stress, compression, stretching forces, and matrix stiffness, all of which contribute to their mechanosensitive response [148]. Several studies have demonstrated the synergistic effects of multiple external and internal mechanical stimuli. Reinwald et al. demonstrated this by investigating the synergistic effects of intermittent hydrostatic pressure (IHP) and topographical fiber alignment on MSC differentiation [148]. Their findings showed that when 270 kPa, 1 Hz hydrostatic pressure was applied to MSC-seeded electrospun polycaprolactone (PCL) nanofiber scaffolds, osteogenic marker expression and calcium deposition were significantly higher in randomly aligned fibers compared to aligned fibers under the same conditions. This suggests that nanotopography and hydrostatic pressure interact to amplify MSC mechanotransduction, with random fibers enhancing focal adhesion dynamics and cytoskeletal reorganization.

Similarly, Ravichandran et al. developed a biomimetic fetal rotation bioreactor that applied biaxial rotation along the X and Z axes, mimicking the gyroscopic motion of a fetus in utero, while simultaneously subjecting MSC-seeded PCL-β-tricalcium phosphate (PCL-TCP) scaffolds to cyclic compression [149]. This dynamic loading system significantly increased MSC proliferation, RUNX2 and COL1A1 expression, and mineralized matrix deposition compared to uniaxial rotation or compression alone. The authors proposed that biaxial rotation enhances fluid transport and nutrient exchange, while cyclic compression reinforces integrin clustering and cytoskeletal tension, collectively amplifying mechanotransduction signaling pathways. These findings emphasize that multimodal mechanical stimulation better replicates the in vivo bone microenvironment, making it an effective strategy for bone tissue engineering. Another critical interplay exists between viscoelasticity and cyclic loading. Yang et al. examined the combined effects of hyaluronic acid-based viscoelastic hydrogels and dynamic compression (5%, 1 Hz) on MSC osteogenesis [150]. Their results revealed that fast-relaxing (highly viscoelastic) hydrogels combined with cyclic compression significantly enhanced RUNX2, ALP, and OPN expression, compared to either stimulus alone. Further, in a rat calvarial defect model, scaffolds with viscoelastic stress relaxation and mechanical loading exhibited superior bone formation, demonstrating that tunable viscoelasticity amplifies the osteogenic response to mechanical loading.

Beyond external mechanical stimulation, internal mechanical properties such as substrate stiffness and surface topography play an essential role in directing MSC fate. Ribeiro et al. studied the combined influence of substrate stiffness and surface topography on human bone marrow stem cells (hBMSCs) cultured on biodegradable polymeric substrates [151]. Their findings revealed that osteogenic differentiation was significantly enhanced on substrates with higher stiffness (12 kPa) and anisotropic surface topography, as evidenced by increased calcium deposition and alkaline phosphatase (ALP) activity. The authors further demonstrated that grooved surface topographies enhanced focal adhesion maturation, amplifying osteogenic signaling. Conversely, the softer 7 kPa substrates tended to favor tenogenic and adipogenic differentiation, emphasizing that stiffness plays a crucial role in MSC lineage commitment. Additionally, Hou et al. explored the synergistic effects of surface roughness and substrate stiffness using hydrogels with tunable roughness gradients (200 nm to 1.2 μm) and stiffness levels (3.8 kPa to 31.3 kPa) [152]. Their results revealed that highly rough but soft hydrogels exhibited enhanced osteogenesis, with upregulated RUNX2 and ALP expression (Figure 2), comparable to or exceeding that observed on smooth, stiff substrates. The study demonstrated that rougher surfaces promoted integrin clustering, leading to increased focal adhesion maturation and YAP nuclear translocation, thereby enhancing mechanotransduction. Interestingly, while soft hydrogels with high roughness facilitated substrate deformation and ligand recruitment, stiff substrates showed optimal osteogenic differentiation at intermediate roughness levels (~645 nm), beyond which excessive roughness impaired cell adhesion. These findings suggest that coordinating roughness and stiffness is crucial for optimizing biomaterial design for bone regeneration.

The synergistic effect of stiffness and viscoelasticity is another key consideration in biomaterial design for MSC differentiation and bone regeneration. For example, Prouvé et al. systematically investigated the combined effect of substrate stiffness (15–140 kPa) and stress relaxation (15–70%) on MSC osteogenesis [46]. Their findings revealed that a stiff matrix (140 kPa) with high stress relaxation (70%) significantly enhanced RUNX2 expression, collagen deposition, and mineralized matrix formation compared to matrices with low relaxation or lower stiffness. The study demonstrated that fast-relaxing stiff matrices facilitated integrin clustering, focal adhesion maturation, and YAP/TAZ nuclear translocation, collectively promoting osteogenic differentiation. Interestingly, while stiffness alone provided a strong mechanical cue, the presence of viscoelastic stress relaxation amplified cellular mechanotransduction. Similarly, Zhang et al. reviewed hydrogels with tunable stiffness and viscoelasticity, emphasizing that mimicking the dynamic mechanical properties of the ECM better simulates the in vivo environment [153]. By using controlled crosslinking strategies, including ion bonding, hydrogen bonding, and supramolecular interactions, they found that dynamic matrix properties influence MSC adhesion, proliferation, and differentiation, underscoring the importance of tuning both stiffness and viscoelasticity in scaffold design.

For the synergistic effect of porosity and stiffness, Ghouse et al. investigated the combined effects of scaffold stiffness and porosity on bone regeneration using additively manufactured porous titanium scaffolds [154]. Their findings demonstrated that higher porosity improved nutrient diffusion and cell infiltration, whereas stiffness influenced MSC mechanotransduction and bone matrix deposition. Importantly, scaffolds with intermediate stiffness-porosity balance exhibited the most effective osteogenic response, as excessive stiffness impaired mechanosensing, while high porosity weakened mechanical integrity. Further, Arabnejad et al. explored high-strength porous biomaterials with different architectural topologies (Tetrahedron and Octet truss structures) [155]. Their findings revealed that scaffolds with an intermediate porosity (~60%) and optimized truss architecture achieved the best osteogenic outcomes by balancing mechanical integrity with biological function. While high porosity improved cellular infiltration, excessive porosity weakened load-bearing capacity, whereas overly stiff structures restricted cell adhesion and differentiation. These results emphasize that co-designing porosity and mechanical properties in scaffold fabrication can enhance bone regeneration while maintaining biomechanical compatibility. The combination of multiple physicomechanical stimuli more effectively mimics the native bone environment and enhances MSC differentiation and bone regeneration.

Across physicomechanical strategies, the effectiveness of a scaffold depends on how well its mechanical and structural properties match the biological requirements of bone healing. Matrix stiffness can promote osteogenic mechanotransduction, but excessive stiffness may impair cell infiltration, cause stress shielding, or induce unfavorable immune responses. Viscoelasticity and stress relaxation provide a more dynamic mechanical environment that can regulate cell spreading, matrix remodeling, and lineage commitment beyond stiffness alone. Surface topography and ligand presentation influence focal adhesion formation and cytoskeletal tension, whereas pore size and porosity regulate nutrient transport, vascular invasion, and tissue ingrowth. Dynamic forces such as shear stress, compression, and vibration can further enhance osteogenesis by mimicking the mechanically active environment of bone. These findings suggest that physicomechanical design should not focus on one isolated property, but should coordinate stiffness, relaxation, architecture, degradation, and loading to support both early cell recruitment and later matrix mineralization.

### 3.4. Mechanotransductive Signaling in Biomaterial-Guided Osteogenesis

Mechanical cues from biomaterial scaffolds include intrinsic material properties, such as stiffness, viscoelasticity, surface topography, and porosity, as well as externally applied or locally generated dynamic forces, including fluid shear stress, cyclic stretch, compression, and vibration. MSCs sense these mechanical signals through multiple mechanosensitive structures at the cell-material interface, including integrins and focal adhesions, cytoskeletal networks, mechanosensitive transcriptional regulators such as YAP/TAZ, and ion channels such as Piezo1/2 and TRPV4 [156,157]. These mechanosensors do not function as isolated pathways. Instead, they form an interconnected signaling network that converts scaffold-derived physical cues into biochemical signals and ultimately regulates osteogenic transcriptional programs [40].

Integrin-mediated adhesion is one of the central mechanisms by which MSCs sense biomaterial mechanics [158]. Scaffold stiffness, surface topography, and adhesive ligand presentation promote integrin clustering and focal adhesion assembly, leading to activation of FAK/Src signaling and downstream RhoA/ROCK-mediated actomyosin contractility [159]. Increased cytoskeletal tension promotes stress fiber formation, nuclear deformation, and nuclear localization of YAP/TAZ. In parallel, FAK/Src signaling can activate MAPK cascades, including ERK1/2, JNK, and p38, which regulate RUNX2 phosphorylation, stability, and transcriptional activity [93]. Therefore, integrin/FAK, RhoA/ROCK, MAPK, and YAP/TAZ signaling provide a major mechanotransductive axis linking scaffold mechanics to osteogenic differentiation [160]. From previous papers discussed, stiff hydrogel substrates have been shown to enhance osteogenic differentiation by increasing ERK1/2 phosphorylation, promoting YAP nuclear localization, and upregulating osteogenic markers such as RUNX2 and OCN [122,124]. These findings suggest that stiffness-driven osteogenesis is not mediated by a single pathway, but by coordinated activation of focal adhesion signaling, cytoskeletal tension, MAPK activity, and nuclear mechanotransduction [119,120,123].

Mechanosensitive ion channels provide another important route through which dynamic mechanical forces regulate MSC osteogenesis [161]. Fluid shear stress, membrane deformation, cyclic strain, and matrix loading can activate Piezo1/2 and TRPV4, resulting in intracellular Ca^2+^ influx [162,163]. Calcium signaling subsequently activates calcium-dependent pathways, including calmodulin, calcineurin/NFAT, CaMK, and MAPK signaling. These pathways interact with YAP/TAZ and Wnt/β-catenin signaling to amplify osteogenic transcription. For instance, Piezo-mediated Ca^2+^ influx can activate calcineurin, which promotes nuclear activation of NFAT, YAP, and β-catenin [164]. Similarly, TRPV4-mediated Ca^2+^ signaling has been linked to mechanically induced RUNX2 activation and matrix mineralization in MSCs [157]. Thus, dynamic forces such as shear stress and cyclic loading can stimulate osteogenesis by simultaneously engaging ion-channel-mediated Ca^2+^ signaling and adhesion-mediated mechanotransduction [146].

Importantly, mechanical signaling also interacts with classical biochemical osteogenic pathways. Wnt/β-catenin signaling can be regulated by mechanical loading, cytoskeletal tension, and YAP/TAZ activity, leading to enhanced β-catenin stabilization and transcriptional activation of osteogenic genes [160]. BMP/Smad signaling, although typically considered a biochemical pathway, is also influenced by the mechanical microenvironment [165]. Matrix stiffness, cell spreading, and cytoskeletal organization can modulate BMP responsiveness, Smad activity, and BMP-related osteogenic gene expression. Conversely, biochemical cues such as adhesive peptides and ECM-mimetic ligands regulate integrin engagement and focal adhesion formation, thereby influencing how cells perceive matrix stiffness, topography, and viscoelasticity [40,158]. These interactions demonstrate that biochemical and mechanical signaling pathways are highly interconnected during biomaterial-guided osteogenesis.

Together, these pathways converge on key osteogenic transcriptional regulators and downstream matrix genes. RUNX2 acts as an early master regulator of osteogenic commitment, while OSX/SP7 functions downstream of RUNX2 to promote osteoblast maturation [40]. Mechanical cues can enhance RUNX2 activity through several routes, including FAK/MAPK-mediated phosphorylation, YAP/TAZ nuclear translocation, Wnt/β-catenin activation, and Ca^2+^/NFAT signaling [166]. These convergent pathways promote the expression of early osteogenic markers such as ALP, matrix-associated genes such as COL1A1, and later osteoblast markers such as OCN and osteopontin [167]. Therefore, biomaterial-guided MSC osteogenesis should be understood as the result of pathway convergence rather than the activation of isolated mechanotransduction cascades.

From a design perspective, these mechanistic interactions suggest that osteogenic biomaterials should be engineered to coordinate multiple mechanical inputs rather than optimize a single parameter. Increasing matrix stiffness or adhesive ligand density can enhance integrin/FAK signaling and cytoskeletal tension, but excessive stiffness may impair cell spreading, remodeling, or immune compatibility [168,169]. Viscoelasticity and stress relaxation can regulate how cells dynamically sense matrix resistance over time, influencing cytoskeletal remodeling, YAP/TAZ activity, and matrix deposition [144]. Micro- and nanoscale topographies can spatially organize focal adhesions and cytoskeletal architecture, while pore architecture and perfusion can generate fluid shear stress that activates Piezo1/2- and TRPV4-dependent Ca^2+^ signaling [127,134,135]. Dynamic mechanical loading can further integrate integrin-mediated force transmission with ion-channel-mediated calcium signaling [147]. Thus, effective scaffold design should aim to coordinate stiffness, viscoelasticity, topography, porosity, and mechanical loading to activate convergent mechanotransductive pathways that drive osteogenesis, matrix deposition, and mineralization.

### 3.5. Translational Challenges of Physicomechanical Cues

Although physicomechanical cues offer a compelling alternative to soluble biochemical delivery, their clinical translation is still constrained by several practical and biological challenges. A major limitation is that the mechanical microenvironment of an implanted scaffold is difficult to control once it enters the body, because the actual forces experienced by the construct depend on defect location, fixation method, patient activity, tissue coverage, and the rate of host remodeling [40]. In addition, the mechanical properties that are beneficial immediately after implantation may not remain optimal throughout healing, since bone regeneration is a dynamic process in which scaffold stiffness, porosity, and load transfer evolve over time. As a result, a scaffold that provides appropriate early mechanical stimulation may later become mechanically mismatched if its degradation, deformation, or pore remodeling proceeds too rapidly or too slowly [170]. This makes it difficult to define a single “optimal” mechanical design for all stages of repair.

Another translational challenge is that many physicomechanical cues are highly context dependent and difficult to standardize across preclinical models. For example, scaffold architecture, pore geometry, and local strain fields can strongly influence osteogenic outcomes, but these same parameters may behave differently in small rodent defects versus large load-bearing defects in sheep, goats, or other clinically relevant models [170]. In vivo delivery of mechanical stimulation also faces engineering and regulatory hurdles, especially when external actuation, magnetically responsive systems, or dynamically loaded implants are required to generate biologically effective forces [171]. Although these approaches can improve osteogenesis, their clinical translation is often complicated by concerns related to device safety, reproducibility, patient compliance, and manufacturing scale-up [172,173,174,175,176,177]. Moreover, because skeletal healing is influenced by both mechanics and biology, models that optimize initial strain alone may still fail to support long-term regeneration if vascularization, immune responses, and tissue integration are not also addressed [170].

Taken together, these limitations indicate that physicomechanical cues should not be viewed simply as a replacement for biochemical signaling, but rather as a design framework that must be carefully tuned to the healing stage, defect type, and mechanical environment. Bone is intrinsically a load-bearing tissue, and mechanical loading is a fundamental regulator of MSC differentiation, matrix deposition, and remodeling [178]. Therefore, the most promising translational strategies are likely to combine mechanically instructive scaffold architectures with stage-specific biological support, rather than relying on either cue alone [179]. In this sense, physicomechanical design may help overcome some of the shortcomings of biochemical delivery by offering a more stable, localized, and persistent form of instruction, but successful clinical adoption will require better control over loading conditions, scaffold degradation, and the evolving bone microenvironment.

**Table 2:** Biomaterial-Induced Osteogenesis Through Physicomechanical Cues.

Physical or Mechanical Properties	Stem Cell Source	Biomaterials	Outcomes	Refer.
**Stiffness**	hMSCs	3D superelastic SiO_2_ nanofibers with chitosan bonding (SiO_2_ NF-CS)	MSCs on stiffened SiO_2_ NF-CS scaffolds showed enhanced FAK/YAP signaling, upregulated osteogenic markers (RUNX2, ALP, OCN), and increased mineralization, promoting osteogenic differentiation and bone formation.	[124]
	hMSCs	PLLA ultrafine fibrous substrates	Stiffer PLLA fiber scaffolds enhanced RUNX2, ALP, and OCN expression via MIF-AKT-YAP signaling, promoting MSC osteogenic differentiation	[123]
	hMSCs	Supramolecular GHG hydrogels	Dynamic hydrogels with a locally stiffened network promote the in situ regeneration of bone defects	[122]
	hBMSCs	GelMA hydrogels	Stiffer GelMA hydrogels enhanced BMMSC osteogenesis by activating PCK2-mediated glycolysis, leading to increased RUNX2 expression and bone regeneration.	[119]
	hBMSCs	CF hydrogel with dual crosslinking	MSCs cultured on the semi-flexible hydrogel exhibited enhanced osteogenesis due to its gradual stiffness transition mimicking native tissue.	[121]
	Mouse MSCs	Alg-Gel composite hydrogels	The softer matrix induces the adipogenic differentiation of MSCs, while the stiffer matrix induces the osteogenic differentiation of MSCs. YAP signaling played a key role in stiffness-mediated lineage commitment.	[120]
**Viscoelasticity**	Murine MSCs	Alginate-based hydrogels	MSCs in rapidly relaxing hydrogels showed enhanced osteogenesis, mineralization, and type I collagen deposition, mimicking natural bone formation.	[127]
	hBMSCs	3D-printed poly(L-lactide) (PLLA) scaffold infused with chitin whisker/chitosan composite hydrogel (CG)	ECM-mimetic viscoelasticity enhanced BMSC adhesion, migration, and osteogenesis by activating integrin-mediated mechanotransduction.	[129]
	hBMSCs	Ionically crosslinked alginate hydrogel	MSC spheroids in viscoelastic hydrogels exhibited enhanced osteogenesis, with increased calcium deposition and ALP activity. The dynamic stress relaxation of the hydrogel promoted MSC differentiation and improved bone formation.	[128]
**Topography**	hMSC	Silicon (Si) nanopillar arrays	The nanopillar arrays are found to enhance osteogenic differentiation of hMSCs	[134]
	hMSC	Polyimide (PI) with grooved/nanopatterned surfaces	Microgrooved surfaces promoted osteogenic differentiation on 2 µm ridges and adipogenic differentiation on 15 µm ridges. Nanogrooved surfaces (650 nm periodicity) enhanced both osteogenesis and adipogenesis, suggesting topography-mediated lineage specification via focal adhesion regulation.	[133]
	hMSCs	HAp/P(VDF-TrFE) piezoelectric composite scaffolds	Promoted bone regeneration, osteogenic differentiation, and cell proliferation via enhanced piezoelectric and topographical cues.	[132]
	hBMSCs	Radially aligned electrospun PCL nanofiber scaffolds	The scaffolds guided endogenous bone regeneration and significantly enhanced bone volume and mineral density.	[136]
	hBMSCs	Hydroxyapatite bioceramics with micro-patterned/nanorod hybrid structures	The hybrid surface promoted osteogenesis synergistically by activating integrin-BMP2 signaling and enhancing gap-junctional cell communication.	[135]
**Porosity**	Rabbit BMSCs	3D-printed lotus root-like akermanite (Ca_2_MgSi_2_O_7_) bioceramic scaffolds	Multichannel biomimetic scaffolds significantly enhanced osteogenesis, angiogenesis, and cell proliferation compared to traditional solid scaffolds by improving nutrient and oxygen diffusion.	[140]
	hMSCs	Sr-HT Gahnite bioactive glass-ceramic scaffold	Hexagonal architecture with ~60–70% porosity significantly enhanced compressive strength, fatigue resistance, and load-bearing capability comparable to human cortical bone.	[139]
**Compressive stress**	Mouse MSCs	Octacalcium phosphate–gelatin (OCP/Gel) scaffold	Cyclic compressive strain (~20% deformation) significantly enhanced osteogenic differentiation and osteogenic marker expression (OPN, OCN). Excessive strain inhibited differentiation.	[144]
**Tensile stress**	Rat BMSCs	Collagen-coated substrates	Cyclic uniaxial tensile strain significantly promoted proliferation and osteogenic differentiation by upregulating bone-related genes (Cbfa1/Runx2, ALP, IGF-II, TGF-β).	[145]
**Fluid shear stress**	Rat BMSCs	3D microporous LTMC polyester scaffolds	Subphysiological laminar fluid flow alone induced osteogenic differentiation and increased collagen type I synthesis, ALP activity, and calcium deposition without osteogenic supplements.	[146]
**Vibration**	hMSCs	Collagen gels cultured in nanovibrational bioreactor	Nanovibrational stimulation (30 nm amplitude, 1 kHz) significantly promoted osteogenesis and increased osteogenic markers	[147]
**Hydrostatic pressure & topography**	hBMSCs	Electrospun aligned/random PCL nanofibers	Hydrostatic pressure (270 kPa, 1 Hz) combined with random nanofibers significantly enhanced osteogenesis and increased osteogenic markers compared to aligned fibers and static controls.	[148]
**Vibration & Cyclic compression**	hMSCs	3D-printed PCL–β-TCP scaffolds	Combined cyclic compression (0.22%, 1 Hz) and biaxial rotation significantly enhanced osteogenic differentiation, gene expression (RUNX2, OCN), and matrix deposition compared to static conditions.	[149]
**Cyclic compression & viscoelasticity**	Rat BMSCs	Sodium alginate–gelatin viscoelastic hydrogel	Cyclic compressive loading (1.5% strain, 1 Hz) combined with fast stress-relaxation viscoelastic hydrogels significantly enhanced osteogenesis through TRPV4-Wnt/β-catenin signaling.	[150]
**Stiffness &** **topography**	hBMSCs	PGCL (7 kPa) and PLTMC (12 kPa) substrates with anisotropic grooves	Stiffer (12 kPa) substrates with anisotropic grooved topography significantly enhanced osteogenesis, increasing calcium deposition and ALP activity.	[151]
**Stiffness &** **roughness**	hMSCs	GelMA hydrogels with nano-to-micro roughness gradients and tunable stiffness	Soft hydrogels with high roughness (1.08 µm) significantly promoted MSC osteogenesis, comparable to stiff substrates, via enhanced integrin-mediated mechanotransduction.	[152]
**Stiffness &** **viscoelasticity**	hBMSCs	Poly(acrylamide-co-acrylic acid) hydrogels with tunable stiffness and stress relaxation	Higher stiffness (~60 kPa) with greater stress relaxation (70%) significantly enhanced osteogenic differentiation and promoted osteocyte maturation.	[46]

## 4. Osteoimmunomodulation: Macrophage–MSC Crosstalk in Biomaterial-Guided Osteogenesis

Bone regeneration is not only regulated by biochemical and physicomechanical cues, but also by the local immune microenvironment [180,181]. Following bone injury or scaffold implantation, macrophages are among the first immune cells recruited to the defect site and play a central role in coordinating inflammation, tissue remodeling, angiogenesis, and osteogenesis [182]. Rather than acting as simple inflammatory cells, macrophages dynamically regulate the healing process through phenotype switching and paracrine communication with MSCs [183]. Therefore, osteoimmunomodulation has emerged as an important design principle for next-generation bone-regenerative biomaterials.

Macrophage phenotypes are commonly simplified into pro-inflammatory M1 and anti-inflammatory or pro-regenerative M2 states, although in vivo macrophage behavior exists along a broader and more dynamic spectrum [184]. During the early stage of bone healing, M1-like macrophages contribute to debris clearance, pathogen defense, and recruitment of reparative cells by secreting inflammatory cytokines and chemokines [185]. However, prolonged or excessive M1 activation can inhibit MSC osteogenesis, increase tissue damage, and contribute to chronic inflammation or fibrous encapsulation. In contrast, timely transition toward M2-like macrophages supports resolution of inflammation, angiogenesis, matrix remodeling, and osteogenic differentiation. M2 macrophages can secrete pro-regenerative factors such as TGF-beta, VEGF, BMP-related factors, IGF-1, and IL-10, which enhance MSC migration, survival, osteogenic commitment, and bone matrix deposition [186,187,188,189]. Thus, successful bone repair requires a balanced immune response rather than simple suppression of inflammation.

The communication between macrophages and MSCs is bidirectional. Macrophages regulate MSC behavior through soluble cytokines, extracellular vesicles, and cell-contact-mediated signaling, while MSCs can also modulate macrophage phenotype through immunoregulatory factors such as prostaglandin E2, TGF-beta, IL-6, IL-10, and indoleamine 2,3-dioxygenase [190]. This reciprocal interaction creates a feedback loop in which the immune response influences MSC-mediated bone formation, and MSCs simultaneously help resolve inflammation and promote tissue repair [183]. Therefore, successful bone regeneration requires not only the activation of osteogenic signaling pathways in MSCs, but also the establishment of an immune microenvironment that supports the transition from inflammation to regeneration [183].

Because macrophage–MSC crosstalk is strongly regulated by local biochemical signals, biomaterials can be engineered to deliver immunomodulatory cues that guide macrophage polarization and indirectly support MSC osteogenesis [191]. This strategy is particularly relevant because bone repair normally requires a staged immune response, with early inflammatory signaling that recruits reparative cells followed by a timely transition toward a pro-regenerative phenotype. Immunomodulatory cytokines such as interferon-γ (IFN-γ), interleukin-4 (IL-4), and interleukin-10 (IL-10) can therefore be used to program macrophage behavior within scaffolds [192]. Spiller et al. demonstrated this concept using decellularized bone scaffolds designed for sequential cytokine delivery [193]. IFN-γ was physically adsorbed onto the scaffold for rapid release to promote an early M1-like macrophage response, whereas IL-4 was attached through biotin-streptavidin binding to provide a more sustained signal supporting later M2-like polarization. In vitro, IFN-γ-releasing scaffolds enhanced M1-associated gene expression, while IL-4-releasing scaffolds promoted M2-associated markers and cytokine secretion over longer culture periods. Although the combined IFN-γ/IL-4 system showed partial overlap between the two signals, in vivo implantation demonstrated that IFN-γ-releasing scaffolds increased CD31+ vascularization compared with control scaffolds. This study illustrates that biochemical scaffold functionalization can be used not only to stimulate osteogenic pathways directly, but also to coordinate macrophage-mediated inflammation and vascularization during tissue repair. Other biochemical factors, including BMP-2, TGF-β, VEGF, bioactive ions, and peptides, may further regulate macrophage behavior by altering cytokine secretion, angiogenic signaling, and the balance between inflammation and regeneration [194]. Therefore, biochemical design should be considered an important strategy for creating a local immunological niche that supports MSC recruitment, osteogenic differentiation, and matrix deposition.

Beyond biochemical signaling, macrophage phenotype is also highly sensitive to physicomechanical cues provided by the surrounding biomaterial matrix. Scaffold stiffness, dimensionality, pore architecture, surface topography, ligand presentation, and degradation behavior can influence macrophage adhesion, cytoskeletal organization, cytokine secretion, and polarization state [195]. Importantly, a biomaterial property that directly promotes MSC osteogenesis may not necessarily create a favorable immune microenvironment. A representative study by He et al. demonstrated that matrix stiffness can regulate MSC osteogenesis through both direct mechanobiological effects and indirect macrophage-mediated immunomodulation [196]. In this study, mouse BMMSCs and RAW264.7 macrophages were cultured in three-dimensional transglutaminase-crosslinked gelatin hydrogels with tunable stiffness. Low-, medium-, and high-stiffness TG-gels were generated using 3%, 6%, and 9% gelatin, respectively, with the low-stiffness matrix showing a yield strength of approximately 1.58 kPa and the high-stiffness matrix reaching approximately 60.54 kPa. When BMMSCs were cultured alone, higher matrix stiffness promoted osteogenic differentiation, as indicated by increased Alizarin Red S staining, ALP activity, and upregulation of osteogenic genes including Sp7, Runx2, Alp, OCN, and osteopontin. However, macrophage involvement changed this stiffness-dependent response. Macrophages encapsulated in high-stiffness TG-gels showed stronger M1-like polarization, with increased IL-1β, TNF-α, iNOS, NO, and TNF-α secretion, whereas macrophages in low-stiffness gels exhibited higher M2-associated markers such as Arg, CD206, and IL-10. Conditioned medium and Transwell co-culture experiments further showed that macrophages from low-stiffness gels enhanced BMMSC osteogenesis, whereas macrophages from high-stiffness gels inhibited BMMSC osteogenic differentiation. These findings indicate that a mechanically favorable matrix for MSC osteogenesis may simultaneously generate an unfavorable inflammatory macrophage response, highlighting the need to optimize scaffold stiffness together with immune modulation rather than evaluating MSC responses alone.

In addition to stiffness, matrix viscoelasticity can also regulate macrophage-mediated osteogenesis. Tao et al. recently showed that early regenerated mid-palatal suture tissue in a mouse rapid maxillary expansion model displayed fast stress relaxation, with a relaxation half-time of approximately 8–20 s during days 1–7, while tissue stiffness remained relatively unchanged [197]. This viscoelastic change coincided with increased TGF-β1+F4/80+ macrophages and subsequent MSC accumulation, and macrophage depletion in Lyz2-Cre/iDTR mice impaired new bone formation. To determine whether stress relaxation directly regulates macrophage function, the authors developed ionically crosslinked sodium alginate-PEG hydrogels with similar initial stiffness of approximately 10 kPa but different relaxation rates, including slow-relaxing gels with τ_1/2_ of approximately 20 s and fast-relaxing gels with τ_1/2_ of approximately 8 s. Fast-relaxing hydrogels promoted macrophage spreading, increased M2-associated CD206 and ARG1 expression, reduced M1-associated CD86 and iNOS expression, and enhanced TGF-β1 expression and secretion. Functionally, macrophages cultured on fast-relaxing hydrogels recruited more BMMSCs, and their conditioned medium enhanced BMMSC osteogenesis, as shown by increased ALP activity, mineralization, and ALP and OCN protein expression. In vivo, fast-relaxation hydrogel-macrophage composites promoted greater bone regeneration and Gli1+ MSC accumulation in mouse cranial defect models compared with slow-relaxation or untreated controls. Mechanistically, fast stress relaxation shifted macrophage metabolism from glycolysis toward oxidative phosphorylation by downregulating VASP/HIF1α signaling, thereby supporting a reparative macrophage phenotype. This study highlights matrix viscoelasticity as an important immunomechanical cue linking macrophage metabolism, MSC recruitment, and bone regeneration.

Scaffold pore architecture represents another important physicomechanical regulator of macrophage behavior. Yin et al. compared genipin-crosslinked collagen/chitosan scaffolds with average pore sizes of 160 and 360 μm and found that the larger-pore scaffold more effectively promoted macrophage transition from an early M1-like state toward an M2-like phenotype [198]. Macrophages cultured in 360 μm scaffolds secreted higher levels of pro-regenerative cytokines such as IL-10, TGF-β, and IGF, while reducing M1-associated inflammatory signals. Conditioned medium from these macrophage-scaffold constructs enhanced endothelial tube formation and migration, and in vivo implantation of 360 μm scaffolds resulted in greater VEGF+ cell accumulation and blood vessel formation than 160 μm scaffolds. These findings indicate that pore architecture is an important immunomodulatory parameter that can regulate macrophage polarization and indirectly support angiogenesis and tissue regeneration. Beyond stiffness, viscoelasticity, and pore size, macrophage phenotype can also be influenced by other physicomechanical cues, including surface topography, roughness, fiber diameter and alignment, overall scaffold architecture, dimensionality, and dynamic mechanical loading [195].

Together, these studies demonstrate that osteogenic biomaterial design should consider not only the direct effects of biochemical and physicomechanical cues on MSC osteogenesis, but also their indirect effects on macrophage behavior and macrophage–MSC crosstalk [183]. This consideration is particularly important in real implantation settings, where scaffolds are immediately exposed to a complex inflammatory microenvironment rather than a purified MSC culture system [180]. After implantation, host immune cells, especially macrophages, respond rapidly to the material surface, degradation products, mechanical properties, and local injury signals [199]. These early immune responses can determine whether the scaffold supports constructive remodeling, vascularization, and bone formation, or instead promotes chronic inflammation, fibrous encapsulation, and impaired regeneration [191]. Thus, successful regenerative biomaterials should be designed to coordinate osteogenic cues with appropriate immunomodulatory signals that guide inflammation toward a pro-healing microenvironment.

## 5. Conclusions and Perspectives

Biochemical signaling has long been established as a key driver of MSC osteogenic differentiation and bone regeneration. Growth factors, small molecules, genetic materials, bioactive peptides, cells, and extracellular vesicles can activate essential osteogenic pathways such as BMP/Smad, Wnt/β-catenin, and MAPK signaling (Figure 2), thereby guiding MSC fate, promoting matrix deposition, and enhancing bone formation. Biomaterial-based delivery systems have further improved the efficacy of biochemical cues by enabling localized presentation, controlled release, prolonged retention, and reduced systemic exposure. Despite these advances, biochemical strategies still present important limitations, including rapid degradation or diffusion, inconsistent bioavailability, immune responses, ectopic bone formation, off-target effects, and regulatory concerns. Potent growth factors such as BMP-2 are particularly challenging because high or poorly controlled doses may lead to abnormal bone formation, inflammatory responses, tumorigenic concerns, or neurological complications in anatomically sensitive defects such as spinal bone defects, thereby restricting broader clinical applicability [101,200,201]. Similarly, small-molecule- and gene-based therapies may suffer from instability, variable release behavior, inconsistent cellular uptake, and potential off-target effects, which limit their translational potential. These challenges highlight that future biochemical scaffold design should not simply focus on incorporating osteogenic agents, but should more precisely regulate their dose, timing, localization, release kinetics, and interaction with the surrounding regenerative microenvironment.

Recent advances in biomaterial-based mechanical stimulation provide a complementary strategy for regulating MSC behavior through physicomechanical cues. The native bone ECM not only supplies biochemical signals, but also provides structural and mechanical stimuli, including stiffness, viscoelasticity, topography, porosity, and dynamic forces such as shear stress, compression, stretch, and vibration. These physicomechanical cues regulate cell adhesion, cytoskeletal organization, focal adhesion maturation, nuclear mechanotransduction, and osteogenic gene expression, thereby directly influencing MSC lineage commitment. Mechanistically, scaffold-derived mechanical signals activate interconnected pathways involving integrins, FAK/Src, RhoA/ROCK, MAPK, YAP/TAZ, Piezo1/2, TRPV4, and calcium-dependent signaling, which ultimately converge on osteogenic regulators such as RUNX2 and OSX/SP7 and downstream markers including ALP, COL1A1, and OCN. Compared with soluble biochemical cues that may undergo rapid diffusion or degradation, engineered biomaterials with tunable mechanical properties can provide more stable, localized, and durable regulatory signals. However, individual mechanical parameters such as stiffness or viscoelasticity should not be optimized in isolation. Bone tissue undergoes continuous remodeling under combined compressive, shear, and tensile forces, and reproducing these dynamic conditions in biomaterials may better reflect the in vivo bone healing microenvironment. Nevertheless, the optimal magnitude, timing, and combination of these mechanical cues remain incompletely understood, requiring further investigation into how multiple physicomechanical inputs interact to regulate MSC osteogenic commitment.

In addition to issues related to biochemical bioactivity and physicomechanical regulation, scaffold degradation is a critical determinant of translational success in bone regeneration [202]. Ideally, biomaterials should degrade in coordination with the rate of new bone formation, so that mechanical support is maintained during the early stages of healing and progressively transferred to the regenerating tissue as matrix deposition increases [202]. If degradation occurs too rapidly, the scaffold may lose structural integrity before sufficient bone ingrowth has occurred, thereby compromising defect stability and repair [203]. Conversely, overly slow degradation may delay remodeling, limit tissue integration, hinder the replacement of the scaffold by newly formed bone, and potentially prolong foreign-body responses. Importantly, degradation should be considered together with changes in porosity, mechanical retention, vascular invasion, and the biological effects of degradation products, since these factors jointly influence the regenerative microenvironment [204]. For example, in a long-term rabbit femoral defect study, Huang et al. compared fast-degrading n-HA/PLGA, slowly degrading n-HA/PCL, and non-degradable n-HA/PA66 scaffolds and found that although the PLGA scaffold initially produced more new bone at 3 months, its rapid degradation caused loss of structural integrity and a subsequent decline in bone volume. In contrast, the PCL and PA66 scaffolds maintained structural integrity and supported continued bone formation over 12 months [205]. Therefore, advanced biomaterials should be designed so that degradation kinetics, mechanical durability, porosity evolution, vascularization, and bone ingrowth proceed in a coordinated and stage-appropriate manner across the healing timeline.

Osteoimmunomodulation has also emerged as a critical design axis for bone-regenerative biomaterials. The immune microenvironment, particularly macrophage–MSC crosstalk, strongly influences inflammation resolution, angiogenesis, MSC recruitment, osteogenic differentiation, and matrix remodeling [180,190]. Successful fracture healing requires carefully coordinated communication between inflammatory cells and bone-forming cells, and impaired macrophage activity can disrupt normal bone repair. During the healing process, macrophages undergo dynamic phenotypic transitions. M1-like macrophages dominate the early inflammatory phase, contributing to debris clearance and secretion of proinflammatory cytokines that recruit reparative cells to the injury site. In contrast, M2-like macrophages emerge later and secrete anti-inflammatory and pro-regenerative mediators that support osteogenesis, angiogenesis, matrix remodeling, and MSC-driven tissue formation. M2-associated macrophages can produce osteogenic and angiogenic cytokines, including BMP-2, TGF-β, VEGF, IGF-1, and IL-10, which directly or indirectly enhance MSC osteogenic differentiation and bone matrix deposition [206]. Conversely, excessive or prolonged M1-dominated inflammation can impair bone repair, emphasizing the importance of a balanced and temporally regulated immune response. MSCs themselves also actively participate in this immune regulation; for example, transplanted MSCs have been shown to promote a shift from M1-like to M2-like macrophage phenotypes, creating a more osteogenic and pro-healing local microenvironment [207]. Thus, next-generation scaffolds should be designed not only to stimulate MSC osteogenesis directly, but also to guide macrophage behavior and immune remodeling toward constructive tissue repair.

Moving forward, smart biomaterials that dynamically adapt their biochemical, mechanical, degradative, and immunomodulatory properties in response to cell-mediated remodeling could further enhance regenerative outcomes. Advances in electrospinning, 3D printing, biofabrication, dynamic hydrogels, and computational modeling offer new opportunities to create scaffolds with precisely controlled architecture, mechanics, porosity, degradation, and release behavior. AI-driven predictive modeling may further accelerate biomaterial design by identifying optimal combinations of biochemical, physicomechanical, and immunomodulatory cues for patient-specific applications [208,209]. However, translational challenges must be addressed to bridge the gap between promising in vitro or small-animal results and clinical application. Scaffold scalability, reproducibility, sterilization compatibility, mechanical durability, long-term safety, and regulatory approval remain major barriers to widespread clinical adoption. The regulatory pathway for smart or multi-stimuli biomaterials is especially complex when scaffolds incorporate growth factors, gene delivery vectors, cells, extracellular vesicles, ion release, degradation-responsive signaling, or mechanically adaptive behavior, which may classify them as combination products requiring evaluation of both device-related and biologic/drug-related risks [210,211]. This issue is particularly relevant for osteoinductive morphogenetic factors such as recombinant BMP-2 [101]. FDA-approved rhBMP-2 bone graft products have specific contraindications and warnings, including restrictions in skeletally immature patients, pregnant women, patients with active infection, and patients with hypersensitivity to product components [200,201]. These regulatory limitations reflect broader concerns that poorly controlled osteoinductive signaling may cause ectopic bone formation, inflammation, abnormal tissue growth, or other local complications when dose, localization, or release kinetics are not tightly controlled [212]. Therefore, clinically translatable osteogenic scaffolds must be designed with well-defined composition, reproducible manufacturing, sterilization-compatible bioactivity, controlled release kinetics, predictable degradation products, immune compatibility, long-term mechanical safety, and validation in clinically relevant preclinical models [213].

**Figure 3:**
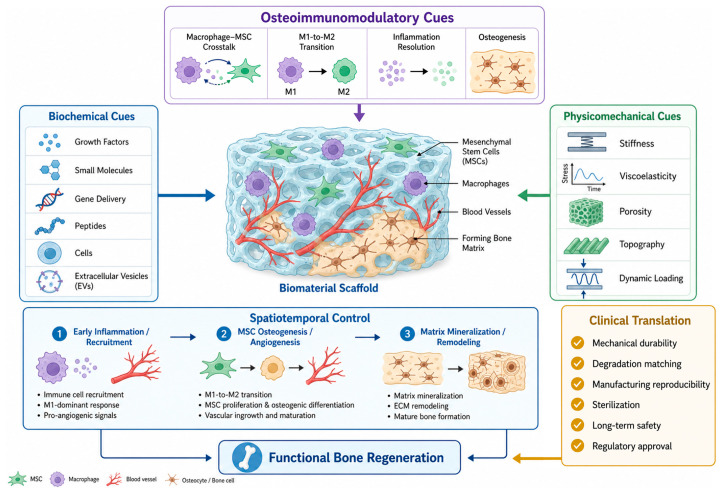
Integrated design framework for next-generation osteogenic biomaterials (figure generated partially with the assistance of Perplexity).

Overall, the most promising biomaterials for bone regeneration are unlikely to rely on a single osteogenic cue. Instead, future scaffolds should function as integrated microenvironment-regulating systems that coordinate biochemical signaling, physicomechanical regulation, osteoimmunomodulation, vascularization, degradation, and tissue maturation in a spatiotemporal manner. An ideal bone-regenerative scaffold would support early immune recruitment and vascularization, promote MSC osteogenic commitment during the regenerative phase, guide matrix deposition and mineralization, and maintain sufficient mechanical integrity until newly formed bone can assume load-bearing function. By shifting from single-cue optimization to coordinated microenvironment design, next-generation biomaterials may better replicate the natural bone healing sequence and provide safer, more effective, and more clinically translatable strategies for functional bone regeneration (Figure 3).

Ideal osteogenic biomaterials integrate biochemical, physicomechanical, and osteoimmunomodulatory cues with spatiotemporal control. Biochemical cues promote MSC osteogenesis, physicomechanical cues regulate mechanotransduction and matrix remodeling, and osteoimmunomodulatory cues guide macrophage–MSC crosstalk, inflammation resolution, and angiogenesis. Coordinated control of these cues across early inflammatory recruitment, intermediate osteogenesis/angiogenesis, and late mineralization/remodeling supports functional bone regeneration. Clinical translation further requires mechanical durability, degradation matching, reproducible manufacturing, sterilization compatibility, long-term safety, and regulatory approval. 

## Data Availability

No new data were created or analyzed in this study. Data sharing is not applicable to this article.
